# Multi-functionalization of reduced graphene oxide nanosheets for tumor theragnosis: Synthesis, characterization, enzyme assay, in-silico study, radiolabeling and in vivo targeting evaluation

**DOI:** 10.1007/s40199-023-00487-7

**Published:** 2023-12-11

**Authors:** Tamer M. Sakr, Mohammed F. Elsabagh, Hend Fayez, Mona O. Sarhan, Yasmin M. Syam, Manal M. Anwar, Mohammed A. Motaleb, Wafaa A. Zaghary

**Affiliations:** 1 Radioisotopes Production Facility, Second Egyptian Research Reactor Complex, Egyptian Atomic Energy Authority, Cairo, 13759 Egypt; 2 Radioactive Isotopes and Generator Department, Hot Labs Center, Egyptian Atomic Energy Authority, Cairo, 13759 Egypt; 3 Labeled Compounds Department, Hot Labs Center, Egyptian Atomic Energy Authority, Cairo, 13759 Egypt; 4 Department of Therapeutic Chemistry/ National Research Centre, Cairo, Egypt; 5 Department of Pharmaceutical Chemistry, Faculty of Pharmacy, Helwan University, Cairo, Egypt

**Keywords:** Nanographene sheets, Technetium-99m, Nano synthesis, Tumor imaging, Radiolabeling, Active targeting, In-silico study, PRAP-1 inhibitor, Tumor theragnosis

## Abstract

**Background:**

In this study, a combination of nanotechnology, organic synthesis and radiochemistry were utilized in order to design an efficient nano-system conjugated with a suitable radionuclide and an antitumor agent for possible application as tumor theragnostic agent.

**Method:**

Four novel compounds (3 and 4a-c) bearing tetrahydroquinazoline-7-sulfonohydrazide or 1,2,3,4-tetrahydroquinazoline-7-sulfonamide scaffold were designed. Then, docking study predicted that the compounds can be considered as potential inhibitors for PARP-1. Following that; the four compounds were synthesized and properly characterized using ^*1*^*H*NMR, ^*13*^*C*NMR, IR and Mass spectroscopy. The cytotoxic effect of the four compounds was evaluated against breast cancer cell line (MDA-MB-436), where compound 3 showed the most promising cytotoxic effect. The inhibitory effect of the four compounds was evaluated in vitro against PARP-1.

**Result:**

Carboxylated graphene oxide nanosheets (NGO-COOH) were synthesized by a modified Hummer's method and has size of range 40 nm. The NGO-COOH nanosheets were proven to be safe and biocompatible when tested in vitro against normal human lung fibroblast cells (MRC-5). The prepared NGO-COOH nanosheets were conjugated with compound 3 then radiolabeled with ^99m^Tc to yield ^99m^Tc-NGO-COOH-3 with a radiochemical yield of 98.5.0 ± 0.5%. ^99m^Tc-NGO-COOH-3 was injected intravenously in solid tumor bearing mice to study the degree of localization of the nano-system at tumor tissue. The results of the study revealed, excellent localization and retention of the designed nano-system at tumor tissues with targeting ratio of 9.0.

**Conclusion:**

Stirred a new candidate tumor theragnostic agent that is safe, selective and stable.

**Graphical abstract:**

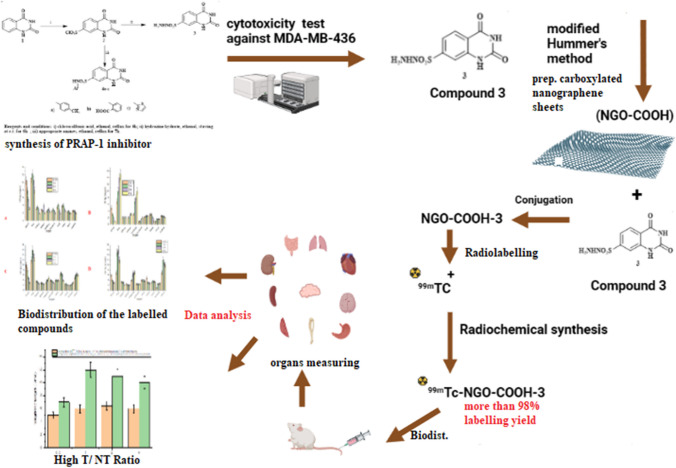

**Supplementary Information:**

The online version contains supplementary material available at 10.1007/s40199-023-00487-7.

## Introduction

The physiology of cancer cells is different than that of the normal cells [1]. Tumor tissues have a completely different vascular system than normal tissue blood vessels [2]. This vascular system in tumor tissue has pervert dynamics, with lineaments like, hyperpermeability and the loss of a basement membrane [3]. When tumor tissues diffusion is constrained, they create new blood vessels for waste removal, and oxygen supply and `this process called Angiogenesis (the formation of new blood vessels) [4]. While enhanced permeability and retention (EPR) effect describes this type of cancer tissue phenomena [2, 5–8].

Due to EPR, nanoparticles (NPs) having diameters up to 100 nm are able to cross the tumor vasculature passively and retained for reasonable time. This fact is what makes NPs excellent carriers for active drugs or a contrast agent [9, 10]. The efficiency of NPs can be further improved by active targeting. This can be achieved via inoculating/ conjugating the NPs with an active drug that has the ability to interact with a specific target which is highly accumulated on the surface of the tumor cells. This technique causes NPs to actively target the tumor tissue, increases their localization, retention, selectively delivers the active agent, improving the therapeutic potential of the conjugated ligand and decreasing undesirable side effects. In this aspect NPs can be conjugated with various tumor specific agents including antibodies, peptides or a chemotherapeutic agent [11]. Various NPs have been utilized in the field of nuclear medicine where the NPs act as a carrier for a chosen radioisotope for the purpose of imaging, therapy or both. ^99m^Tc-chitosan NPs, ^99m^Tc-bovine serum albumin NPs, ^99m^Tc-Aspergillus flavus synthesized copper oxide NPs have been found to be efficient in diagnostic purposes. Meanwhile, ^111^In- multifunctional superparamagnetic iron oxide NPs has found its use as a cancer therapeutic agent [12–17].

Graphene has been explored for different imaging applications due to its unique characteristics, safety and rapid cellular uptake [18]. So, graphene based nanosheets are excellent nano-systems when compared to other NPs [19, 20].

PARP-1, poly(ADP-ribose) polymerase-1, is responsible for repairing the DNA damage within the cell [21]. For that reason, PARP-1 is one of the intriguing targets for cancer therapy where PARP-1 inhibitors showed efficiency in inducing cellular death. Various PARP-1 inhibitors are at clinical investigation for the treatment of various tumors such as olaparip, iniparib, veliparib and talazoparib [22].

**Fig. 1:**
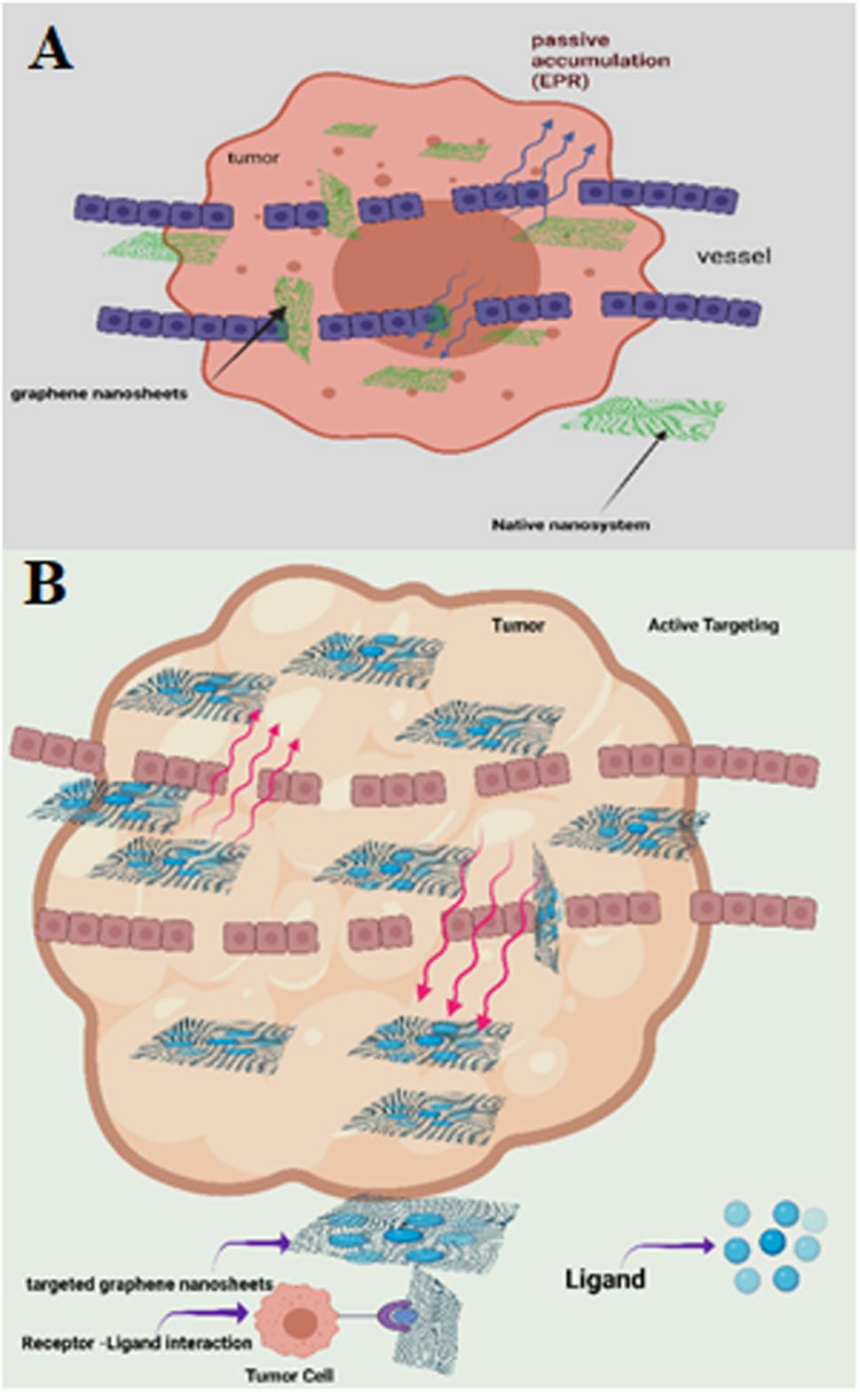
**A** Schematic diagram of passive targeting of graphene oxide (GO) nanosheets; **B** Active targeting of decorated graphene oxide nanosheets

In this work, a novel nano-system comprised of carboxylated nanographene sheets (NGO-COOH) conjugated with both ^99m^Tc and a novel cytotoxic agent is investigated as a radio-theragnostic agent. ^99m^Tc has been chosen for this study due to its excellent characteristics as a radiotracer for use in single-photon emission computed tomography (SPECT) [23–28]. The designed nano-system is expected to target the tumor cells via both passive and active targeting as illustrated in Fig. 1. The designed nano-system will be investigated for its biocompatibility and selective accumulation at tumor site.

## Experimental

### Materials

#### Chemicals

Chloroform, Graphite, ethyl acetate H_2_O_2,_ H_2_SO_4_, KMnO_4_ NaNO_3_ and NaOH were bought from Sigma (St. Louis, Mo., USA). Fetal Bovine serum, DMEM, RPMI-1640, HEPES buffer solution, L-glutamine and gentamycin were purchased from Lonza (Belgium).

#### Radioactive material

Technetium-99 m (^99m^Tc) was eluted as pertechnetate (^99m^TcO_4_^−^) from a ^99^Mo/^99m^Tc generator provided as a gift from Radio-isotopes Production Facility (RPF), Egyptian Atomic Energy Authority (EAEA), Cairo, Egypt.

#### Mammalian cell lines

Normal human lung fibroblast cells (MRC-5) were collected from the American Type Culture Collection (ATCC, Rockville, MD).

### Docking of the synthesized compounds to the Active site of PARP-1

#### Database preparation

All molecules were build using the builder functionality integrated in MOE2015.10 program. The molecules were subjected to stochastic conformational search and energy minimization using MMFF94X forcefield. All the generated conformation were saved as.mdb file to be used for later docking [29].

#### Protein targets preparation

PARP-1 crystal structure was downloaded from the protein data bank depository (https://www.rcsb.org/), PDB 4GV7 [30]. For protein preparation, all included water molecules were removed, 3D protonation was performed, and energy minimization and correction of bonding pattern was applied.

#### Identification of the binding site

The binding site was determined based on the position of the bounded co-crystallized inhibitor using the site finder functionality in MOE.

#### Docking protocol

Semi-flexible docking was applied using MMFF94x as a force field, triangle matcher as placement method and London dG as scoring function. For validation of the docking protocol, the co-crystallized ligand was included in the docked database and re-docked with the test compounds. Also, the docking protocol was done using Affinity dG as a scoring function to validate the results of the docking [29].

### Synthesis of carboxylated nanographene oxide sheets (NGO-COOH)

Graphite oxide suspension was prepared from natural graphite powder using the modified Hummers' process. Exactly 1 g graphite and 1 g NaNO_3_ were added to 50 mL H_2_SO_4_ and the mixture was stirred for 10 min in an ice bath. Following that, the mixture was allowed to warm to room temperature while gradually adding 6 g of KMnO_4_. The formed suspension was stirred in a water bath (35 ℃), then mixed with one hundred milliliter of deionized water (DI) while keeping the temperature under 60 °C. Finally, 6 mL of hydrogen peroxide (30%) diluted in 200 mL deionized water was added to the suspension to solubilize manganese ions and to prevent the suspension from forming residual permanganate. Centrifugation was performed at 6000 rpm for 10 min then the supernatant solution was extracted and centrifuged several times to remove all the remaining acids and salts. The obtained nanographene oxide (NGO) suspension was sonicated for 30 min to obtain a yellow–brown graphene oxide (GO) suspension. Further centrifugation at 2000 rpm for 15 min was performed to dissolve the remaining unexfoliated graphitic platelets and any formed precipitates were eliminated. For carboxylation of NGO, 10 mL NaOH (12 mg/ mL) was added followed by sonication for 2 h at 800 W to convert OH groups to COOH [31–36].

### Characterization of NGO-COOH nanosheets

Various techniques were used to characterize NGO-COOH nanosheets to ascertain their form, size, surface area, chemical composition, and dispersion. Transmission electron microscopy (TEM) with an acceleration voltage of 200 kV (Ted Pella, Redding, CA, USA), and dynamic light scattering (DLS) at an acceleration voltage of 200 kV (Ted Pella, Redding, CA, USA) were used for characterization. The XPS peak was deconvoluted using Gaussian components after a Shirley background subtraction. The O/C atomic ratio of the NGO sheets were evaluated using peak area ratio of the XP Score levels and the sensitivity factor of each element in XPS. Raman spectroscopy was carried out at room temperature using a HR-800Jobin-Yvon equipped with a 532 nm Nd-YAG excitation source. UV–Visible spectrophotometry using visible recording spectrophotometer UV-160A, Shimadzu, Japan and Fourier transformer infrared spectroscopy (FT-IR), (Mattson Instruments, Inc., New Mexico, USA was used. Samples were prepared for TEM measurements by placing 5- 20 µL of NGO-COOH dispersed solution on a Cu grid and then dried under an IR lamp while the sample had been diluted by utilizing the same sample quantity of bi-distilled water for DLS measurements.

### Synthesis and characterization of *2,4-Dioxo-1,2,3,4-tetrahydroquinazoline-7-sulfonohydrazide*

#### Chemistry

**Scheme 1:**
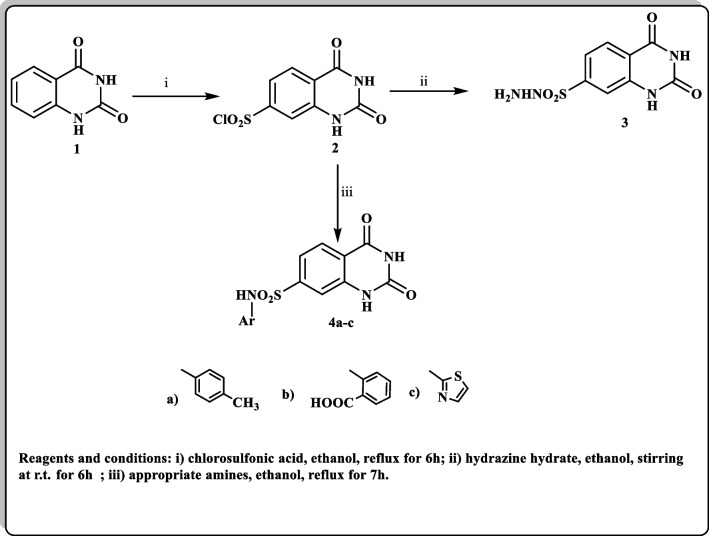
The synthetic approach of the target quinazoline-sulfonohydrazide derivatives (3 and 4a-c)

The synthetic approach of the target quinazoline-sulfonohydrazide derivative as shown in Scheme 1.

##### Preparation of quinazoline-2,4(1H,3H)-dione (1)

Compound **1** was prepared according to reported method [37]. Yield 72%, m.p. > 250 °C.

##### Preparation of 2,4-dioxo-1,2,3,4-tetrahydroquinazoline-7-sulfonyl chloride (2)

Compound **2** was prepared according to reported method. Yield 71%, m.p. 310 °C.

##### 2,4-dioxo-1,2,3,4-tetrahydroquinazoline-7-sulfonohydrazide (3)

To a solution of the sulfonyl chloride derivative **2** (2.60 g, 10 mmol) in ethanol (30 mL), hydrazine hydrate (2 mL, 20 mmol) was added and the reaction was continuously stirred at room temperature for 6 h. The formed precipitate was filtered, washed several times with petroleum ether and then crystallized from ethanol to yield the hydrazide derivative **3** as a white powder.

Yield (65%), m.p.268–270 °C, IR (KBr, cm^−1^): 3344–3320 (4NH); 3132 (CH-aromatic), 2999 (CH-alicyclic); 1750, 1678 (2C = O); 1332, 1138 (SO_2_). ^*1*^*H*NMR (DMSO-*d*_*6*_, *δ* ppm): 4.17 (broad s, 2H, NH_2_, exchangeable with D_2_O); 7.30, 7.9 (2d, 2H, *J* = 7.08 Hz, aromatic-H); 8.28 (s, 1H, aromatic-H); 8.24, 11.31, 11.55 (3 s, 3H, 3NH, exchangeable with D_2_O).^*13*^*C*NMR (DMSO-*d*_*6*_, *δ* ppm): 114.73, 116.54, 128.06, 132.12, 134.23, 144.24, (aromatic-C); 150.61, 162.54 (2C = O). MS, *m/z* (%): 257 [M^+.^ + 1] (30.09), 256 [M^+^] (19.27). Analysis for C_8_H_8_N_4_O_4_S (256.24), Calcd.: %C, 37.50; H, 3.15; N, 21.87; S, 12.51. Found: %C, 37.78; H, 3.37; N, 22.06; S, 12.38.

##### Preparation of 2,4-dioxo-N-substituted-1,2,3,4-tetrahydroquinazoline-7-sulfonamide (4a-c)

To a solution of compound **2** (2.60 g, 10 mmol) in ethanol (30 mL), an appropriate amine namely; *p*-toluidine, 2-aminobenzoic acid and thiazol-2-amine (10 mmol) was added. The reaction mixture was refluxed for 7 h. The formed precipitate was collected by filtration and crystallized from ethanol to give the corresponding derivatives **4a-c**.

##### 2,4-Dioxo-N-(p-tolyl)-1,2,3,4-tetrahydroquinazoline-7-sulfonamide (4a)

Yield (74%), m.p. < 300 °C, IR (KBr, cm^−1^): 3344–3320 (3NH); 3132 (CH-aromatic); 2996 (CH-alicyclic); 1750, 1678 (2C = O); 1332, 1138 (SO_2_). ^*1*^*H*NMR (DMSO-*d*_*6*_, δ ppm): 2.32 (s, 3H, CH_3_); 6.95–8.20 (m, 7H, aromatic-H); 11.21, 11.31, 11.50 (3 s, 3H, 3NH, exchangeable with D_2_O). ^*13*^*C*NMR (DMSO-*d*_*6*_, δ ppm): 20.76 (CH_3_); 121.31, 123.34, 124.51, 126.85, 129.43, 130.12, 130.67, 138.20, 141.37, 142.84, 144.28, 150.50 (aromatic-C); 150.67, 163.11 (2C = O). MS, *m/z* (%): 332 [M^+.^ + 1] (45.09), 331 [M^+^] (41.89). Analysis for C_15_H_13_N_3_O_4_S (331.35), Calcd.: %C, 54.37; H, 3.95; N, 12.68; S, 9.68. Found: %C, 54.57; H, 3.70; N, 12.93; S, 9.90.

##### 2-(2,4-Dioxo-1,2,3,4-tetrahydroquinazoline-7-sulfonamido) benzoic acid (4b)

Yield (74%), m.p. 280–282 °C, IR (KBr, cm^−1^): 3350- 3320 (3NH); 3025 (CH-aromatic), 2905 (CH-alicyclic); 1750, 1710, 1680 (3C = O); 1332, 1135 (SO_2_). ^*1*^*H*NMR (DMSO-*d*_*6*_, *δ* ppm): 6.71–6.90 (m, 2H, aromatic-H), 7.10 (d, 1H, aromatic-H), 7.14–7.26 (m, 1H, aromatic-H); 7.81, 7.87 (2d, 2H, *J* = 7.08 Hz, aromatic-H); 8.20 (s, 1H, aromatic-H), 11.08, 11.21, 11.31 (3 s, 3H, 3NH, exchangeable with D_2_O); 11.55 (s, 1H, OH, exchangeable with D_2_O). ^*13*^*C*NMR (DMSO-*d*_*6*_, δ ppm): 117.10, 123.61, 128.67, 132.43, 134.70, 130.53, 134.69, 137.90, 141.45, 142.84, 144.67, 150.81 (aromatic-C); 150.61, 162.91, 171.11 (3C = O). MS, *m/z* (%): 362 [M^+.^ + 1] (36.50), 361 [M^+^] (28.03), Analysis for C_15_H_11_N_3_O_6_S (361.33), Calcd.: % C, 49.86; H, 3.07; N, 11.63; S, 8.87. Found: % C, 50.09; H, 3.27; N, 11.85; S, 9.17.

##### 2,4-dioxo-N-(thiazol-2-yl)-1,2,3,4-tetrahydroquinazoline-7-sulfonamide (4c)

Yield (74%), m.p. 265–267 °C, IR (KBr, cm^−1^): 3348- 3330 (3NH); 3021 (CH-aromatic); 2910 (CH-alicyclic); 1746, 1680 (2C = O); 1332, 1135 (SO_2_). ^*1*^*H*NMR (DMSO-*d*_*6*_, *δ* ppm): 6.71, 7.11 (2d, 2H, *J* = 7.08 Hz, aromatic-H); 7.21- 8.20 (m, 3H, aromatic-H); 11.08, 11.21, 11.50 (3 s, 3H, 3NH, exchangeable with D_2_O). ^*13*^*C*NMR (DMSO-*d*_*6*_, δ ppm): 124.10, 128.15, 129.63, 131.90, 138.90, 141.45, 142.84, 144.67, (aromatic-C); 154.61, 168.91 (C = O). MS, *m/z* (%): 324 [M^+.^] (28.03). Analysis for C_11_H_8_N_4_O_4_S_2_ (324.33), Calcd.: % C, 40.74; H, 2.49; N, 17.28; S, 19.77. Found: % C, 40.59; H, 2.27; N, 17.65; S, 19.43.

### Evaluation of cytotoxic effects

#### Evaluation of cytotoxic effects of NGO-COOH nanosheets

The in vitro cytotoxic effect of NGO-COOH was tested using Normal human lung fibroblast cells (MRC-5). The cells were grown on RPMI-1640 medium supplemented with 10% inactivated fetal calf serum and 50 µg/mL gentamycin. The cells were maintained at 37 ºC in a humidified atmosphere with 5% CO_2_ and were sub-cultured 2–3 times per week.

For the cytotoxicity assay, the cell lines were suspended in medium at concentration 5 × 10^4^ cells/ well in Corning® 96-well tissue culture plates, then incubated for 24 h. NGO was then added into 96-well plates (three replicates) to achieve twelve concentrations for it. Six vehicle controls with media were run for each 96 well plate as a control. After incubating for 24 h, the numbers of viable cells were determined by MTT test. The 50% inhibitory concentration (IC_50_) was estimated using Graphpad Prism software (San Diego, CA. USA) from graphic plots of the dose response curve for each conc [38, 39].

#### Evaluation of cytotoxic effect of compound 3 and 4a-c

The cytotoxicity assay was performed ***at Department of therapeutic chemistry/ National Research Center***. cytotoxic activity of target compounds **3, 4a-c** was determined via three independent experiments by 3-(4,5-dimethylthiazolyl-2)-2,5-diphenyltetrazolium bromide (MTT) cell proliferation assay against the cell proliferation of human breast cancer MDA-MB-436 cells carrying natural BRCA1 deficient [R].

### In vitro PARP inhibition assay

The in vitro inhibition of PARP-1 was measured using an HT F Homogeneous 96-well PARP Inhibition Assay Kit (Trevigen, Ca# 4690-096–K, Gaithersburg, USA), according to the manufacturer’s protocol. The synthesized compounds were dissolved in DMSO and then serially diluted to the required concentrations with distilled water, keeping the final concentration of DMSO lower than 1%. Olaparib was used as positive control. Fluorescence values under the condition of excitation wavelength (544 nm) and emission wavelength (590 nm) were measured using a multi-well spectrophotometer (Molecular Devices SpectraMax M5 microplate reader, Careforde, Chicago, USA). Then, the standard curve was drawn and the inhibition rate of each test compound was calculated. IC_50_ value of each compound was calculated using GraphPad Prism 6 software [40, 41].

### Conjugation of NGO-COOH with compound 3 (NGO-COOH-3)

NGO-COOH nanosheets were condensed with compound **3** (12 mM) in the presence of N,N′-Dicyclohexylcarbodiimide (DCC) (12 mM) in DMF. Dicyclohexylurea formed was removed by filtration and DMF was removed under vacuum. The residue obtained was washed with water to remove excess of amine and traces of DMF. The residue was then purified by column chromatography using chloroform/ethyl acetate, 80:20 as an eluent and then recrystallized from alcohol [42–44].

### Radiolabeling procedures

The eluted [^99m^Tc]TcO_4_^−^ was reduced from its hepta-oxidation state to enable the formation of the desired complex [^99m^Tc]TcO_4_-NGO-COOH using sodium dithionite [45]. Compound 3 and its conjugate with NGO-COOH nanosheets were radiolabeled with ^99m^Tc as follow;

#### Radiolabeling of compound 3

A volume of 200 μL of freshly eluted ^99m^TcO_4_^−^ (20 MBq) was added to appropriate amount sodium dithionite (5- 25 mg) followed by the addition of different amounts of compound 3 (50- 750 mg) dissolved in 5 mL of DMF. The mixture was incubated for 10- 50 min and the pH was adjusted using the appropriate buffer solutions at 4- 8.

#### Radiolabeling of NGO-COOH-3

A volume of 200 μL of freshly eluted ^99m^TcO_4_^−^ (20 MBq) was added to appropriate amount sodium dithionite (5- 25 mg) followed by the addition of different amounts of NGO-COOH (50- 500 mg). The mixture was incubated for 10- 60 min and the pH was adjusted using buffer solutions at 4–8.

#### Determination of in vitro stability

The in vitro stability of the radiolabeled complexes was studied in saline at 0.5, 2, 4, 6, 8 and 24 h post-incubation. The radiolabeling reactions were kept at 37 °C and a sample from each reaction mixture was withdrawn and the RCY was re-estimated by paper chromatography.

### Determination of the radiochemical yield (RCY)

The RCY was determined using ascending paper chromatography. After the designated time interval, samples of each radiolabeling reaction mixture (200 µL, 20 MBq) were applied on strips of Whatman paper no. 3 (13 cm × 1 cm). The applied samples were allowed to air dry. Two different mobile phases were used for development [46–50]. First, chloroform/ethyl acetate mixture (1:2) was used as a mobile phase to check the percentage of free ^99m^TcO_4_^−^. Second saline, was used to determine the percent of reduced hydrolyzed ^99m^Tc-colloid (RH-^99m^Tc). After complete development, each paper strip was allowed to dry and cut into 1 cm pieces and counted in a well-type NaI (Tl) γ-counter (BLC-20, BUCK Scientific). HPLC was used to ensure that the labeled molecule was present as a single species and to ascertain the complexation yield. HPLC analysis of ^99m^Tc were done by injection of 10 µl, after 0.20 µm Millipore filtration, into the column (C-18 reversed phase column) and UV spectrophotometer detector (SPD-6A) adjusted to the 270 nm wavelength. The column was eluted with mobile phase (water (A) and acetonitrile (B) mixed with 0.1% trifluoroacetic acid as the mobile phase. the flow rate was adjusted to 1 ml/min. Fractions of 1 ml were collected separately using a fraction collector up to 30 and counted in a well-type NaI (Tl) detector connected to a single-channel analyzer [51].

The RCY was calculated as follows;

$$Radiolabeled\;complex\;\% = 100-({free\_}^{99m}Tc\_{{TcO}^{-}}_{4}\%+RH {-}^{99m} Tc\%).$$

### Biodistribution study of radiolabeled complexes

To form a solid tumor, a 0.2 mL of Ehrlich Ascites Carcinoma fluid was administered intramuscularly in the right thigh of female Swiss Albino mice. The animals were well-cared until the tumors became obvious (7- 10 days). The parent tumor line (Ehrlich Ascites Carcinoma) was withdrawn from 7-day-old Swiss Albino donor females and diluted with sterile physiological saline solution to yield 12.5 x 10^6^ cells/mL [52].

The animal study was conducted in accordance with the EAEA Committee on Animal Ethics (EAEA/2020/193) which follows the criteria set upon by the European Community for the use of animals as an experiment.

Biodistribution studies were performed by injecting the solid tumor-bearing mice intravenously with NGO-COOH nanosheets followed by injecting the radiolabeled complexes. The mice were divided into four groups (four mice per group) according to the designated time of dissection. Following the administration of the radiolabeled nanosheets, mice were dissected at 0.5, 1, 2, 4 h post injection (p.i). Blood, solid tumor, and major organs/tissues were collected and wet-weighed. The distribution of the radioactivity in each organ/ fluid was measured ex vivo*,* the radioactivity in each was detected by a gamma-counter (Perkin Elmer) as presented in Fig. 4. The results were expressed as mean percentage injected dose per gram (%ID/g ± SD) [53–55].

## Result and discussion

### Docking of the synthesized compounds to the Active site of PARP-1

Poly (ADP- ribose) polymerases (PARP) are diphetheria toxin like ADP-ribosyltransferase domain proteins that detect DNA single strand breaks and catalyze their repair by adding ADP-ribose units to acceptor proteins to facilitate the DNA repair process. The inhibition of PARP-1 retards the DNA repair process thus, PARP-1 inhibitors act as radiosensitizers and chemosensitizers and used in combination with alkylating agents and radiotherapy in cancer patients [56–58]. Different PARP-1 inhibitors have been developed and currently are in phase 2 and 3 clinical trials including olaparib [59], rucaparib [60] niraparib [61], veliparib [62] and talazoparib [63].

In this study, the docking of compound **3** to the active site of PARP-1 has been performed along with other known PARP-1 inhibitors (olaparib, and talazoparip) to evaluate their affinity to PARP-1.

**Fig. 2:**
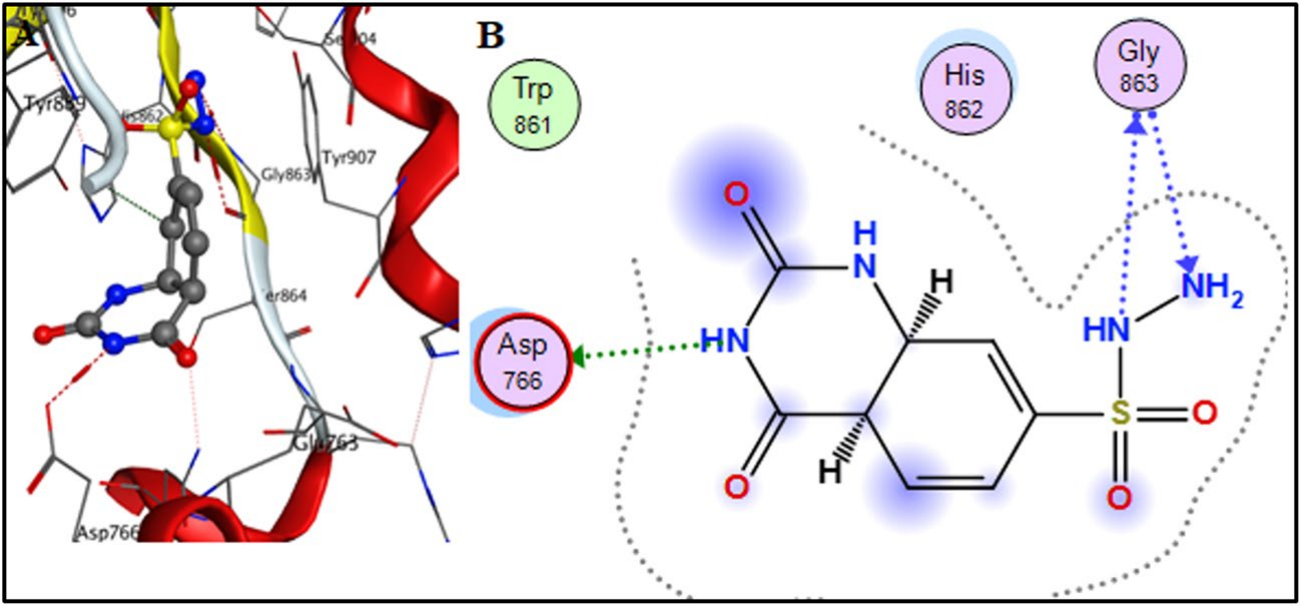
Docking pose of compound 3 the active site of PARP-1; **A** 3D presentation of the docked pose; **B** 2D presentation of the docked pose

Compound **3** showed comparable affinity to the co-crystallized ligand (-6.46 kcal.mol^−1^) as shown in Table 1. Compound **3** secured its fit with the formation of 3 HB interactions with Arg 865, Ser864 and Tyr889 as shown in Fig. 2.

**Table 1:** Docking of compound 3 to the active site of PARP-1

Compound	S (Kcal.mol^−1^)	No of bonds	Amino Acids involved
3	−6.47	3 HB	Arg865, Ser864 and Tyr889
Olaparib	−7.63	1 arene-arene interaction 1 HB	Tyr907 His862
Talazoparib	−6.03	1 HB	Asp766
Co-crystallized	−6.06	3HB	Gly863 and Tyr907

The results of the docking study clearly showed that the suggested compounds had remarkable affinity towards the active site of PARP-1. Compound **3** showed a promising affinity and could be considered as a promising inhibitor for PARP-1 enzyme.

### Chemistry

#### Synthesis of compounds 3 and 4a-c

Synthetic strategy to synthesize the target derivatives **3**, **4a-c** has been illustrated in Scheme 1. The structural formulae of all the new compounds were confirmed by microanalyses and spectral data. Anthranilic acid was stirred with an equivalent amount of urea at 160 °C for 6 h to give the key starting compound 1*H*-quinazolin-2,4-dione **(1)** [37]. Furthermore, compound **1** was treated with chlorosulfonic acid in ethanol to give the corresponding sulfonyl derivative **2**, which was stirred with hydrazine hydrate in ethanol at r.t. f0or 6 h to accomplish the corresponding sulfonohydrazide **3**. Also, compound **2** was allowed to react with different appropriate amines namely; *p*-toluidine, 2-aminobenzoic acid and thiazol-2-amine in refluxing ethanol to afford the corresponding tetrahydroquinazoline-7-sulfonamide derivatives **4a-c**.

#### Synthesis and characterization of NGO-COOH nanosheets

**Fig. 3:**
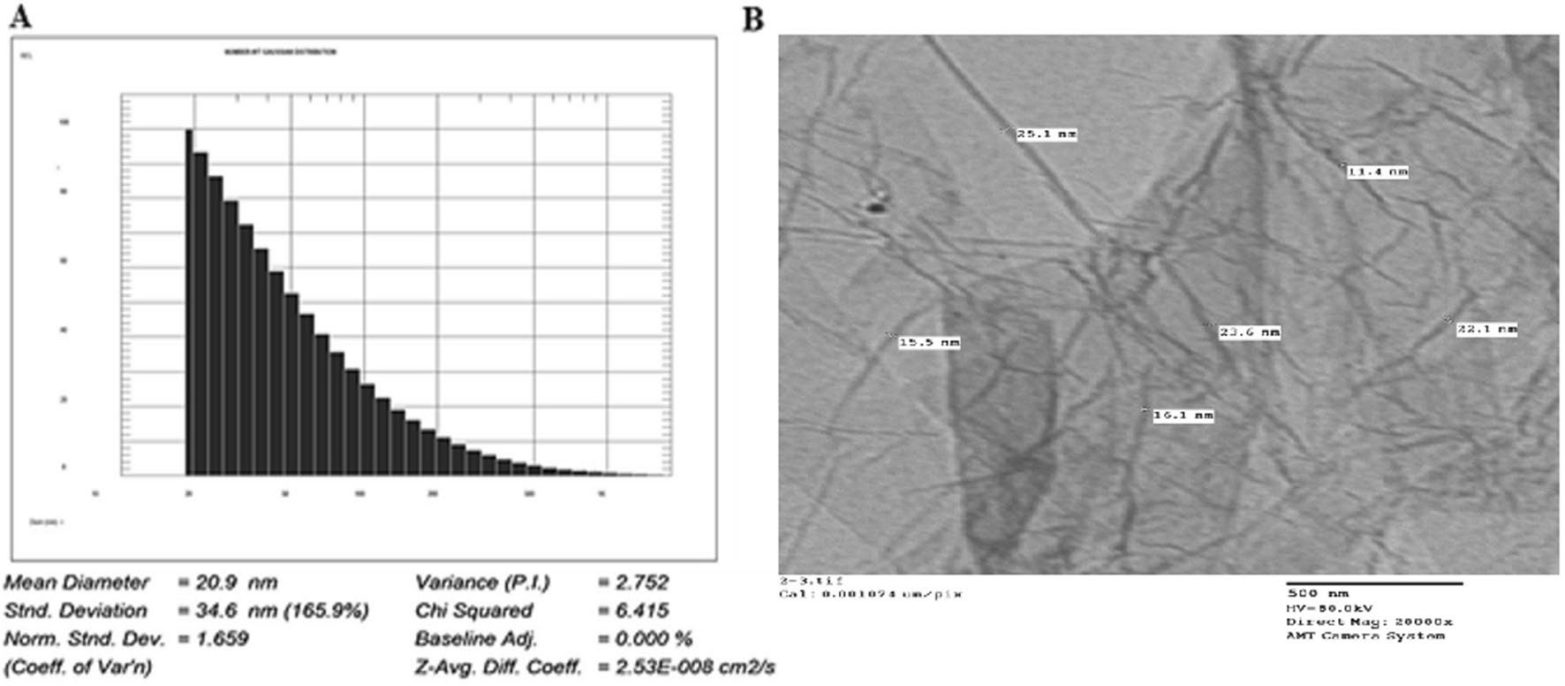
Size analysis of NGO-COOH nanosheets (**A**) DLS scan, **B** TEM image

NGO- COOH nanosheets were synthesized according to the method mentioned earlier. The carboxylation of NGO sheets offer a method for increasing the hydrophilicity of the sheets thus increasing the distance between them. Also, the introduced carboxylic groups act as a reactive site that ease the conjugation of other active molecules such as peptides, enzymes polymers and positively charged molecules [64].When examined by DLS, all small sheets were found to be within the size range of 10–70 nm Fig. 3A. Also, the average diameters of NGO-COOH sheets were 6.5 ~ 70 nm. The shape of NGO-COOH was studied using TEM Fig. 3B. The width of the NGO-COOH sheets was reduced by sonication to less than 100 nm, while their thickness that captured and measured by TEM was 1- 2 nm.

**Fig. 4:**
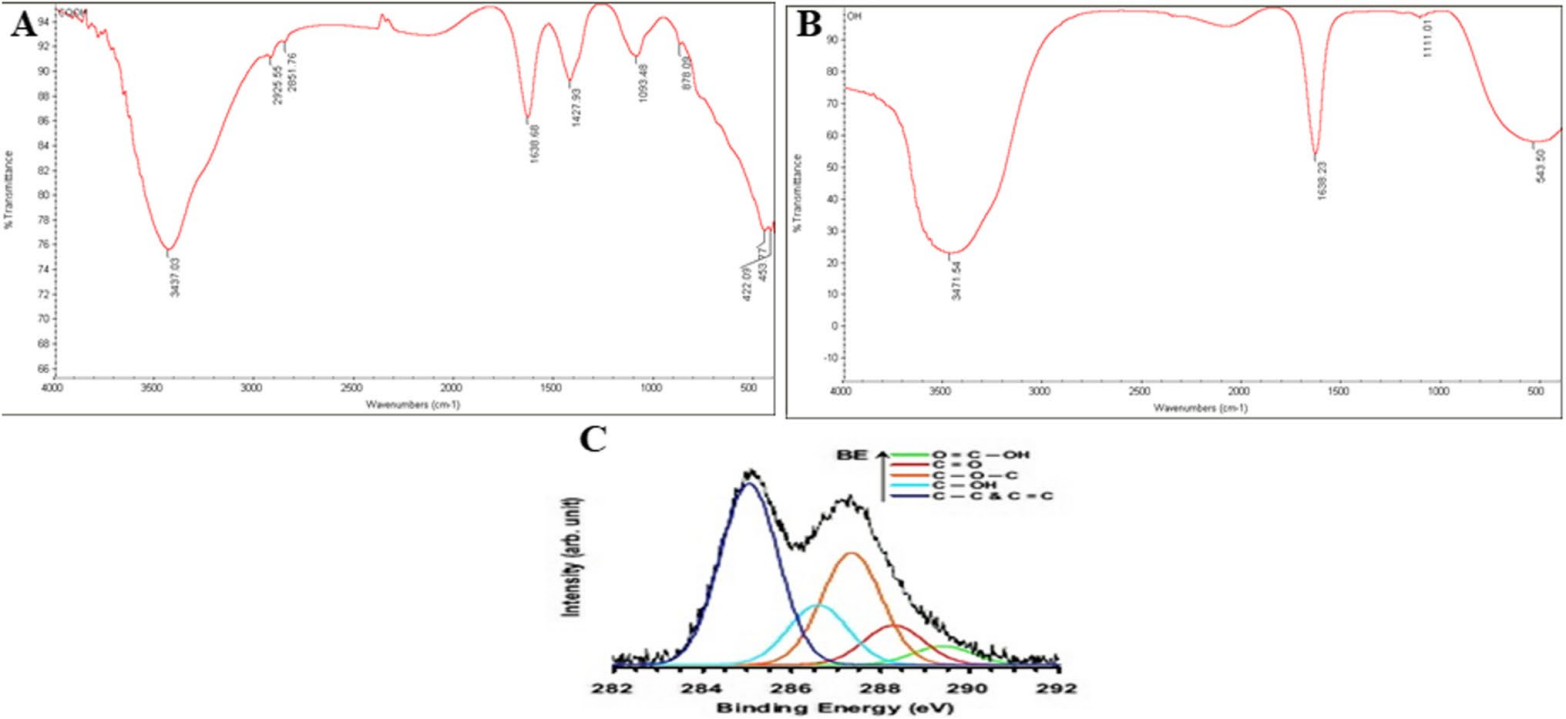
FT-IR spectra of (**A**) NGO-COOH nanosheets, **B** NGO-OH nanosheets and (**C**) XPS spectrum of NGO-COOH nanosheets

FTIR confirmed the existence of COOH groups in the NGO-COOH. As shown in Fig. 4A the appearance of absorption peaks at 3437 (sharp peak) and 1638 cm^−1^ representing C = O group was observed. This peak stretched due to the effect of C = C of cyclic carbon in the hybrid structure of nanographene. On the other hand, FTIR of NG-OH showed a broad band at 3471 cm^−1^ assigned for the OH group as shown in Fig. 4B [65]. Figure 4C represents XPS peak deconvolution of C(1 s) core levels of the GO sheets. In the peak deconvolution, the peak centered at 285 eV was attributed to the C–C and C = C bonds. The other deconvoluted peaks located at the binding energies of 286.6, 287.4, 288.3 and 289.4 eV were assigned to the C-OH, C-O-C, C=O, and O=C-OH oxygen-containing functional groups, respectively [47–49].

**Fig. 5:**
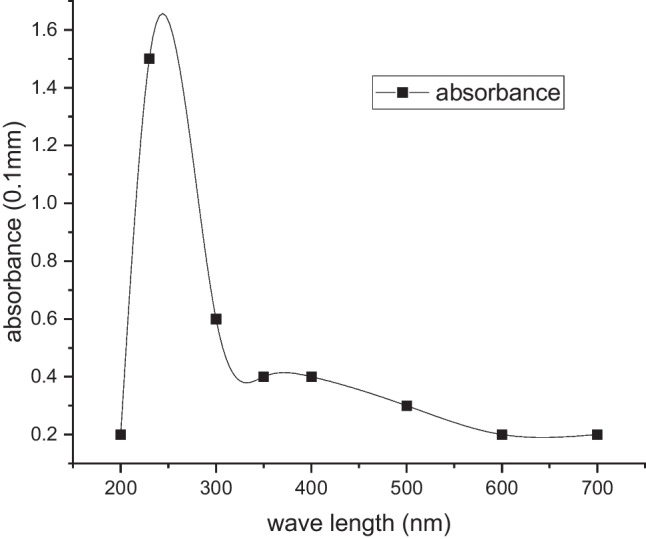
UV/Vis spectra of the NGO-COOH nanosheets

The O/C atomic ratio of the NGO sheets was found to be 0.47. This is consistent with the oxygen content of chemically exfoliated GO sheets reported previously [50, 51]. The COOH groups of NGO were further confirmed by UV/Vis spectroscopy. A peak at 232 nm appeared representing COOH group as shown in Fig. 5. Furthermore, UV/Vis spectrum of NGO sheets presented an absorption peak at 270 nm [31, 65, 66].

#### Synthesis and characterization of NGO-COOH-3 conjugate

**Fig. 6:**
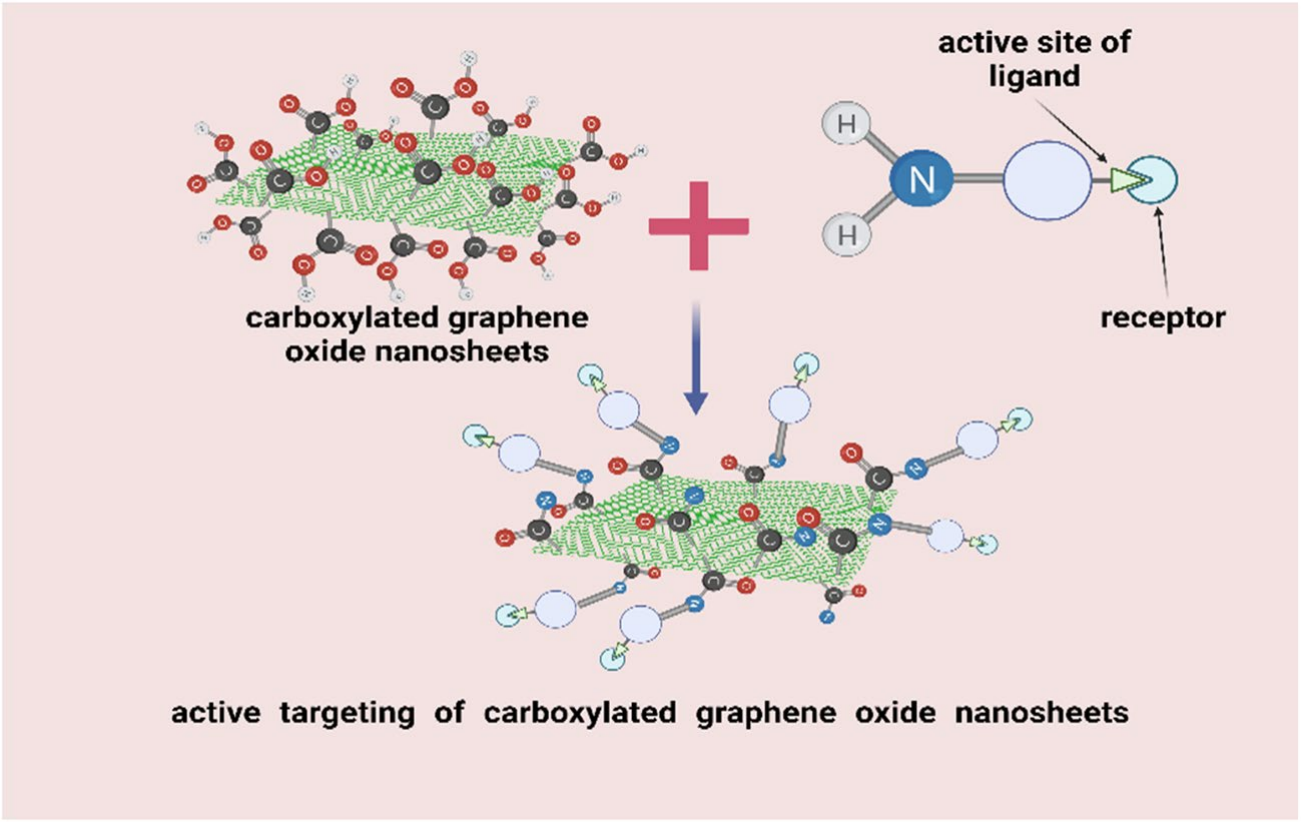
The schematic diagram of the NGO-COOH with compound 3 as active targeting of nanosheets for tumor

Conjugating NGO-COOH nanosheets with compound **3** was intended to actively target the conjugated nano-system to tumor cells. Active targeting will cause increase of the selectivity and retention of the cytotoxic agent (**3**) at tumor site. Thus, enhancing its therapeutic potential (Fig. 6). Compound **3** was chosen from the synthesized compounds based on its promising affinity to PARP-1 as predicted by the docking study and its potent cytotoxic effect on BRCA1 mutant MDA-MB-436 cells.

**Scheme 2:**
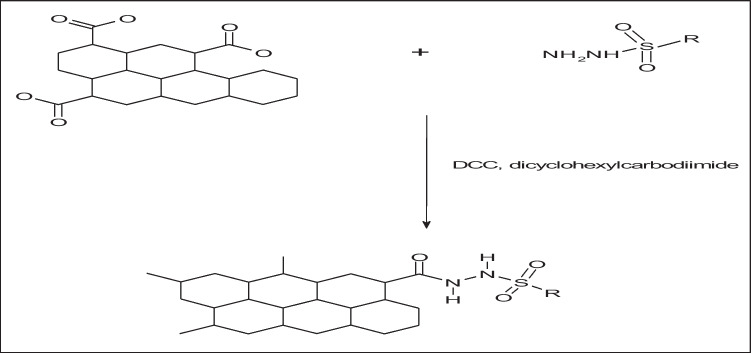
The synthetic approach of the NGO-COOH with compound 3

**Fig. 7:**
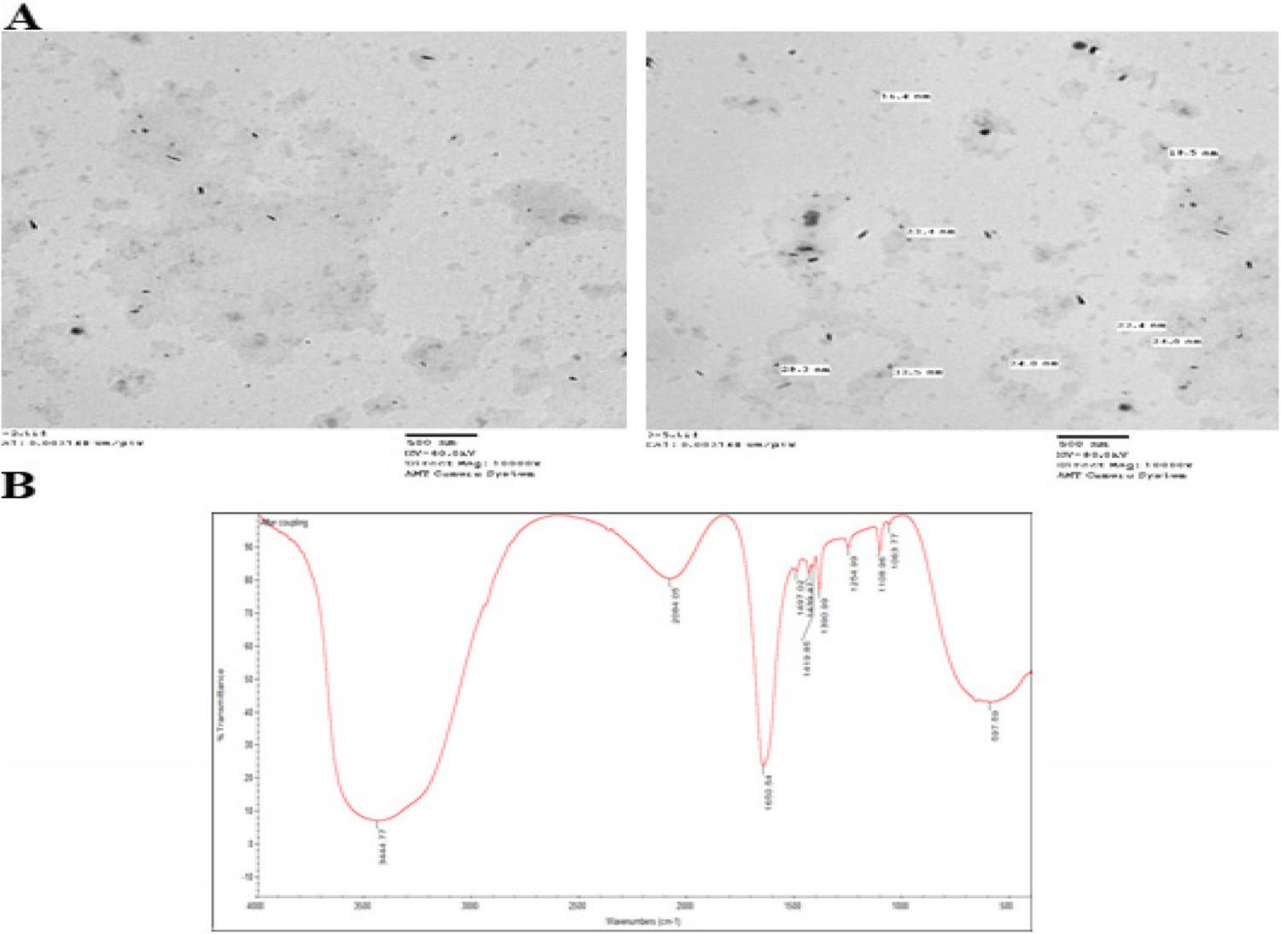
Size analysis of NGO-COOH-3; **A**The FT-IR spectra of NGO-COOH-3 **B**

The conjugation of NGO-COOH nanosheets was performed using the strategy outlined in Scheme 2. Following the conjugation, compound **3** appeared as spots of 25 nm on the sheets of NGO-COOH as measured by TEM and shown in Fig. 7A. The presence of NH of amide group in the complex was also confirmed by FTIR where absorption peaks at 3444 (broad peak) and 1650 cm^−1^were detected representing the C = O, Fig. 7B.

### Cytotoxicity evaluation

#### Cytotoxicity evaluation of the synthesized compounds (3, 4a-c)

The results were expressed in Table 2 as the IC_50_ (μM) values. The hydrazide derivative **3** exhibited the most promising cytotoxic efficiency that was about 1.6 times more potent than that obtained by Staurosporine (IC_50_; 0.006 μM and 0.01 μM, respectively). Compound **4b** displayed equipotent cytotoxic activity to that obtained by Staurosporine of (IC_50_ = 0.01 μM). The significant cytotoxic activity of both analogues 3 and 4b could explained due to the presence of NH_2_ and OH groups which might form additional H-bonds with the target proteins leading to the improvement of the inhibiting effect against the tested cancer cells. On the other hand, a detectable reduction (5, 4 folds) in the potency was observed by the compounds **4a** and** 4c** as shown in Table 2.

**Table 2:** Cytotoxic activity of the new compounds (3 and 4a-c)

Compound No.	IC_50_ (μM) MDA-MB-436
3	0.006 ± 0.1
4a	0.05 ± 0.8
4b	0.01 ± 0.3
4c	0.04 ± 0.8
Staurosporine	0.01 ± 0.1

#### Cytotoxicity evaluation of NGO-COOH nanosheets

**Fig. 8:**
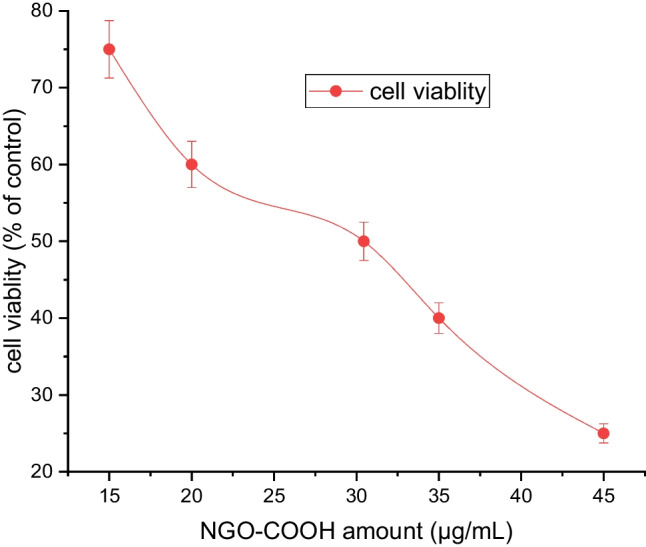
Relative cell viability of NGO-COOH

The cytotoxic effect of NGO-COOH nanosheets was tested normal human lung fibroblast cells (MRC-5) to determine the safety of the nano-system to the body. The determined inhibitory activity of NGO-COOH was found to be 30.45 ± 0.27 µg/mL as shown in Fig. 8. This result illustrates the biocompatibility of NGO-COOH and how much the safety of this organic compound.

### In vitro PARP-1 and NGO-COOH inhibitory assay

The in vitro PARP-1 inhibitory active for the synthesized compounds (**3** and **4a-c)** using olaparib as a reference drug. According to Table 3, the compounds displayed inhibitory activities against PARP-1 with IC_50_ values in the nano-range (49.56- 144.4 ng/mL). However, none of the tested compounds showed potency comparable to olaparib of (IC_50_ = 9.49 ng/mL). It could be noted that; the thiazole derivative **4c** represented the most potent inhibitory effect against the target enzyme of IC_50_ = 49.56 ng/mL followed by compounds **4b** and **3** had IC_50_ of 60.32 and 66.9 ng/mL, respectively. This result indicated that the new compounds their cytotoxic can be attributed to inhibition of PARP-1 along with other mechanism of action. The determined inhibitory activity of NGO-COOH was found to be 30.45 ± 0.27 µg/mL as shown in Fig. 8. This result illustrates the biocompatibility of NGO-COOH and how much the safety of this organic compound.

**Table 3:** In vitro inhibitory activity of the synthesized compounds against PARP-1

Compound No.	IC_50_ (ng/ml) PARP-1
3	66.9
4a	144.4
4b	60.32
4c	49.56
Olaparib	9.49

### Radiolabeling of compound 3 and NGO-COOH-3

**Fig. 9:**
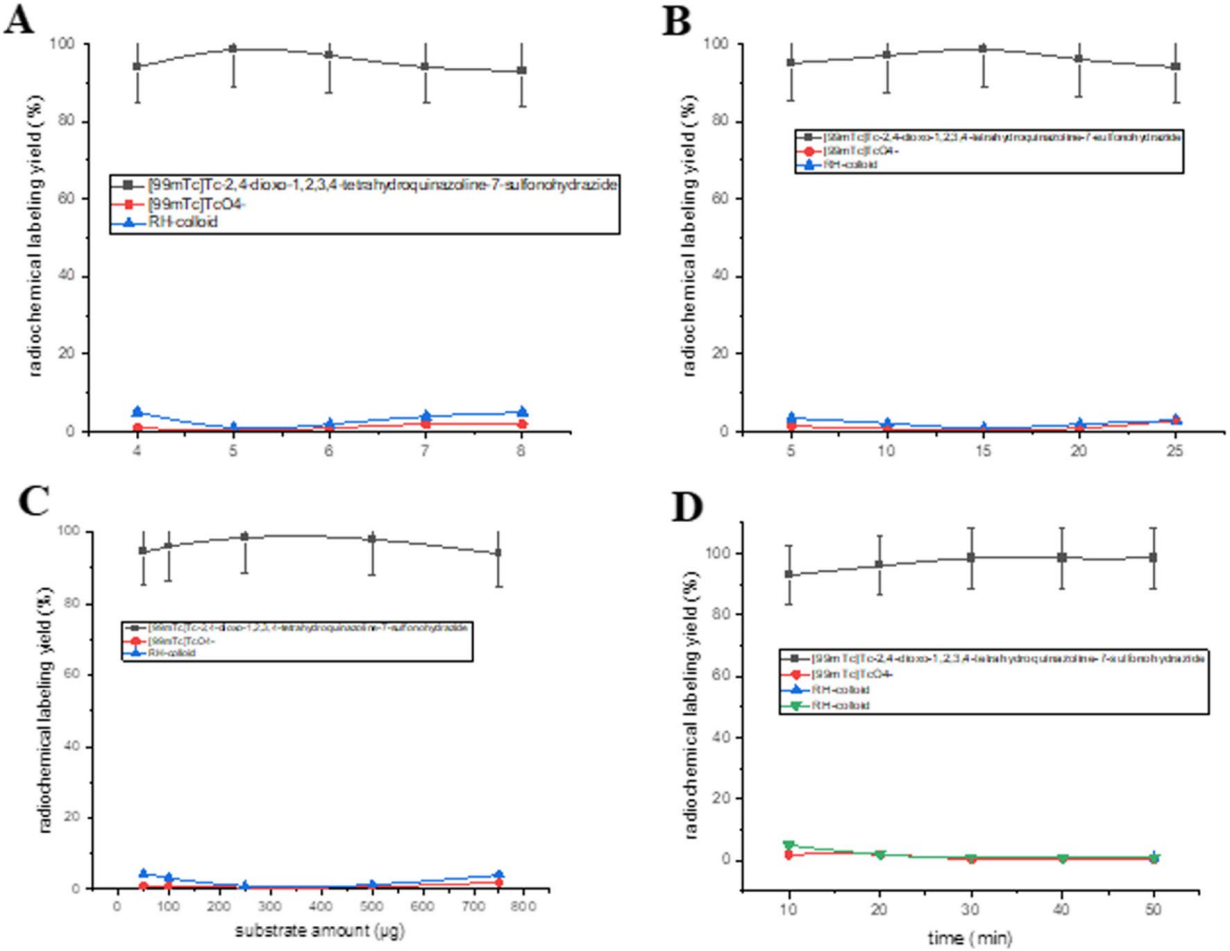
Radiochemical yield of ^99m^Tc-3 (**a**) pH, **b** Sodium dithionite amount, **c** compound 3 amount, **d** reaction time

Radiolabeling compound **3** was planned in order to study its biodistribution profile and the degree of its localization at tumor site. The various conditions affecting the radiochemical yield such as pH of the reaction, reaction time, amount of sodium dithionite and compound **3**. The optimum RCY for obtaining ^99m^Tc-3 was 98.5 ± 0.45% (Fig. 9) using 15 mg of sodium dithionite, 250 mg of compound 3 and the reaction was allowed to proceed for 30 min at pH 5.

**Fig. 10:**
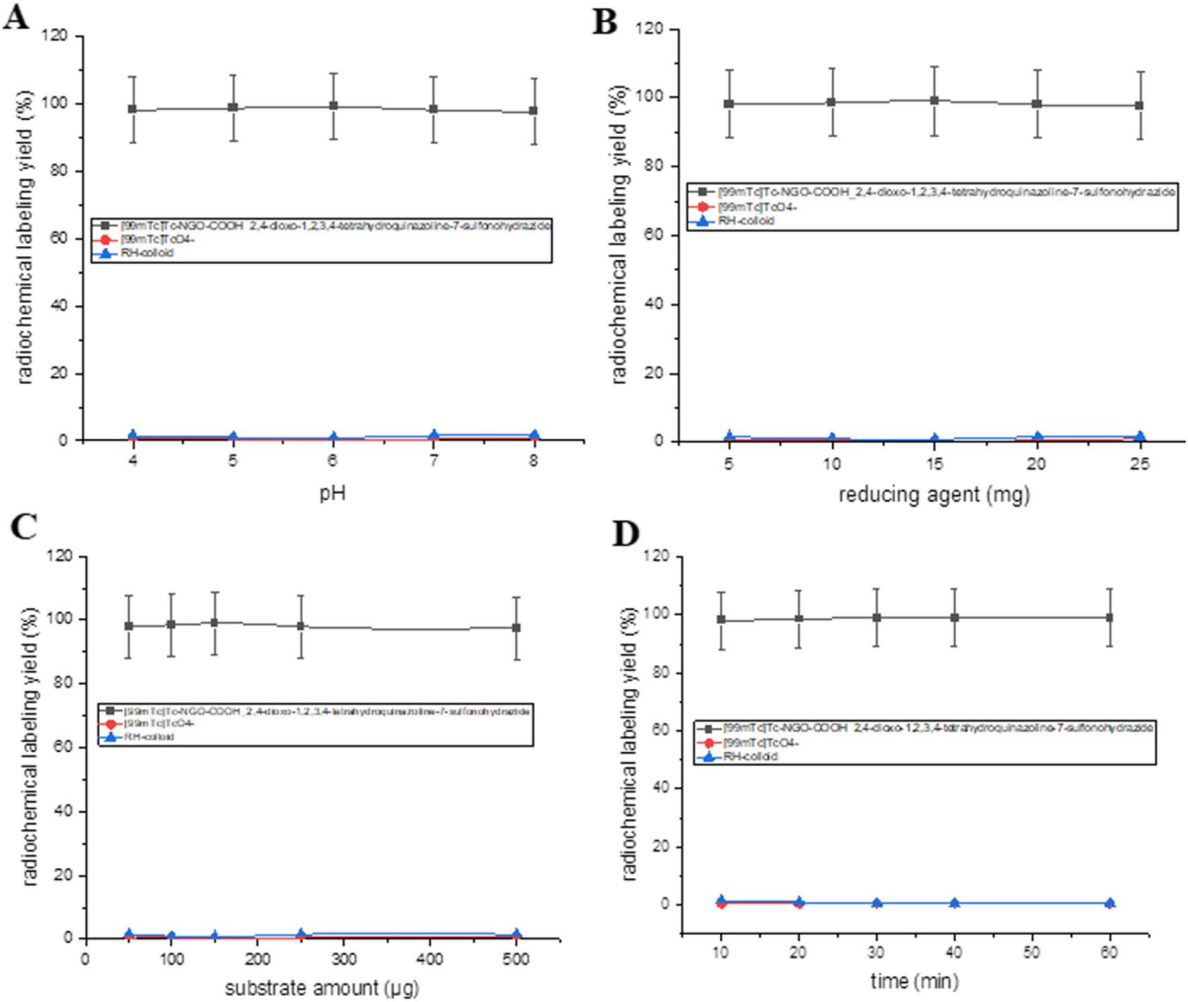
Radiochemical yield of ^99m^Tc-NGO-COOH-3 as a factor of (**a**) pH, **b** reducing agent, **c** substrate amount, **d** reaction time

**Fig. 11:**
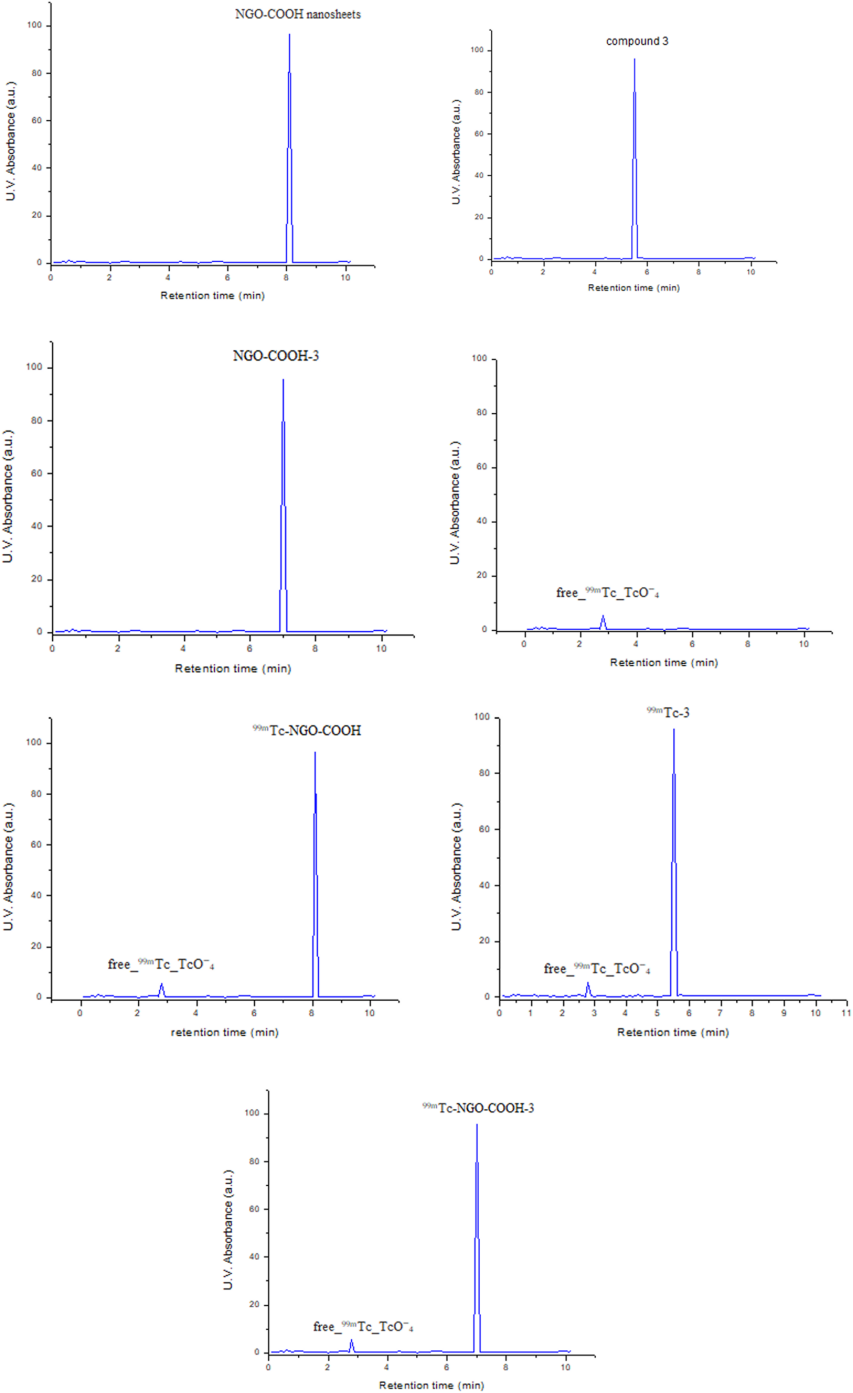
HPLC radiochromatogram and U.V. profile of ^99m^Tc complexes

On the other hand, NGO-COOH-3 was radiolabeled with ^99m^Tc to study the utility of the radiolabeled nano-system for theragnostic use. The optimum RCY for ^99m^Tc-NGO-COOH-3 complex (98.5 ± 0.45%) was obtained using 15 mg sodium dithionite, 150 μg of substrate at pH 6 at reaction time 30 min as shown in Fig. 10. The radiochromatogram was presented in (Fig. 11) and showed two peaks, one at fraction No. 2.8 which corresponds to the free pertechnetate, while the second peak was collected at fraction No. 5.5, 8.1 and 7 that correspond to ^99m^Tc-3, ^99m^Tc-NGO-COOH and ^99m^Tc-NGO-COOH-3 complexes respectively which were found to coincide with the UV signal. The radiochromatogram showed 98.5% labeling yield which was coinciding with the results of the analysis using ascending paper chromatography.

### In vitro stability study

^99m^Tc-3 and ^99m^Tc-NGO-COOH-3 were tested for their in vitro stability in saline to ensure the stability of the radiolabeled complex at time of injection.

**Fig. 12:**
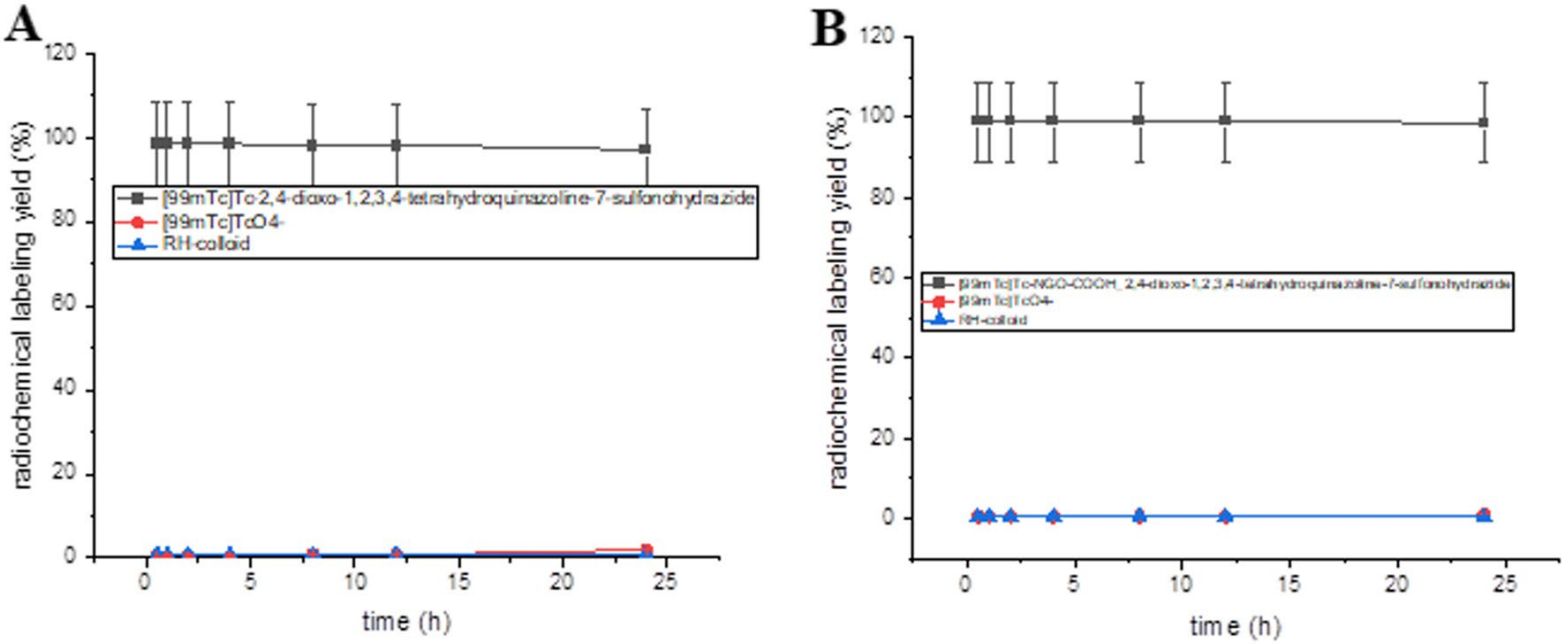
(**A**) ^99m^Tc -3 and (**B**)^99m^Tc—NGO-COOH-3 in vitro stability in saline/serum at 37 °C followed in time

As indicated in Fig. 12, both complexes showed adequate stability observed in high RCY (above 98.5%) for time up to 8 h. Also, after 12 h both complexes were stable (RCY98.5%).

### Biodistribution study

#### Biodistribution of^99m^Tc-3

**Fig. 13:**
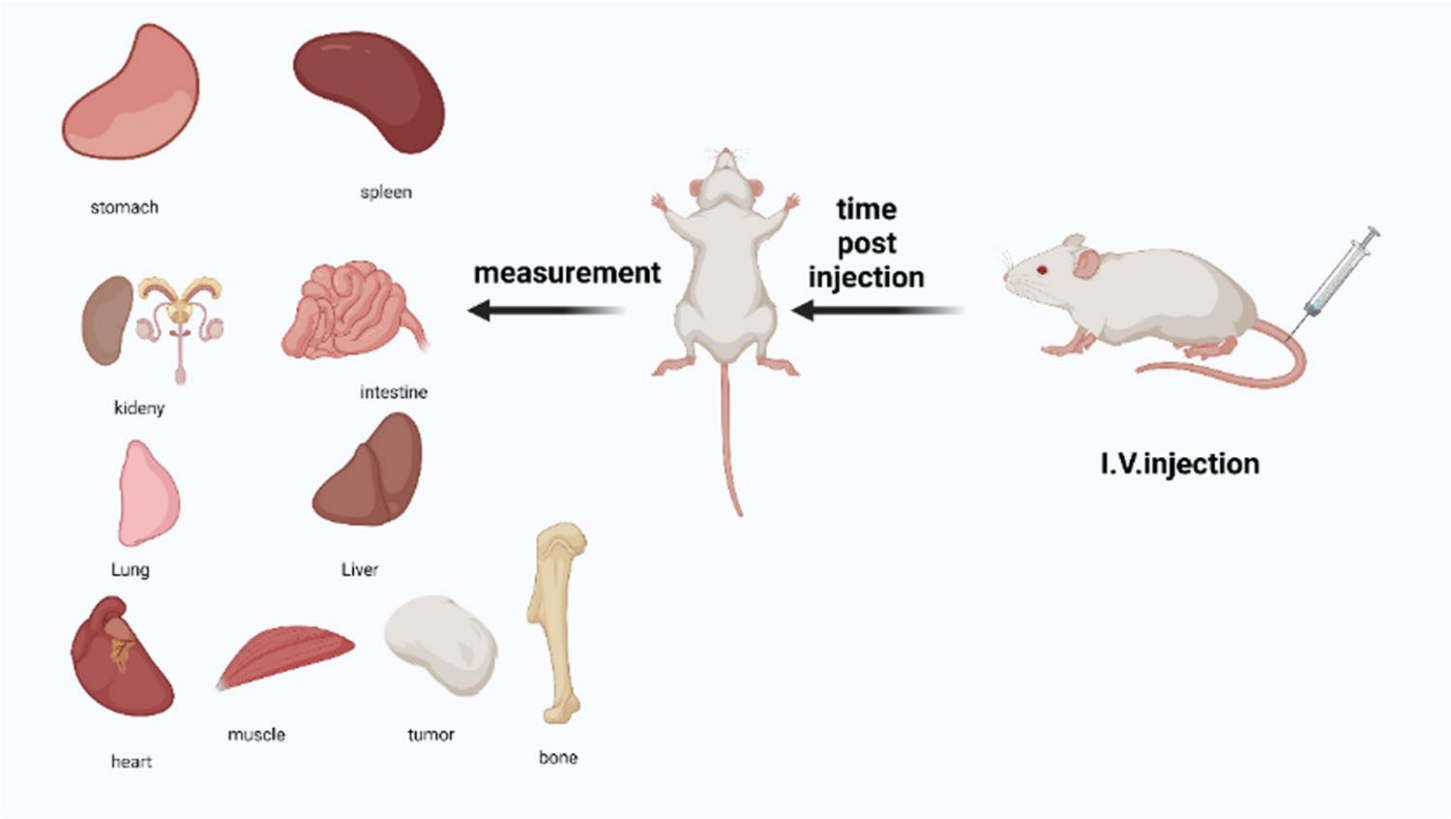
Schematic representation of biodistribution of radiolabeled nanosheets in mice

**Fig. 14:**
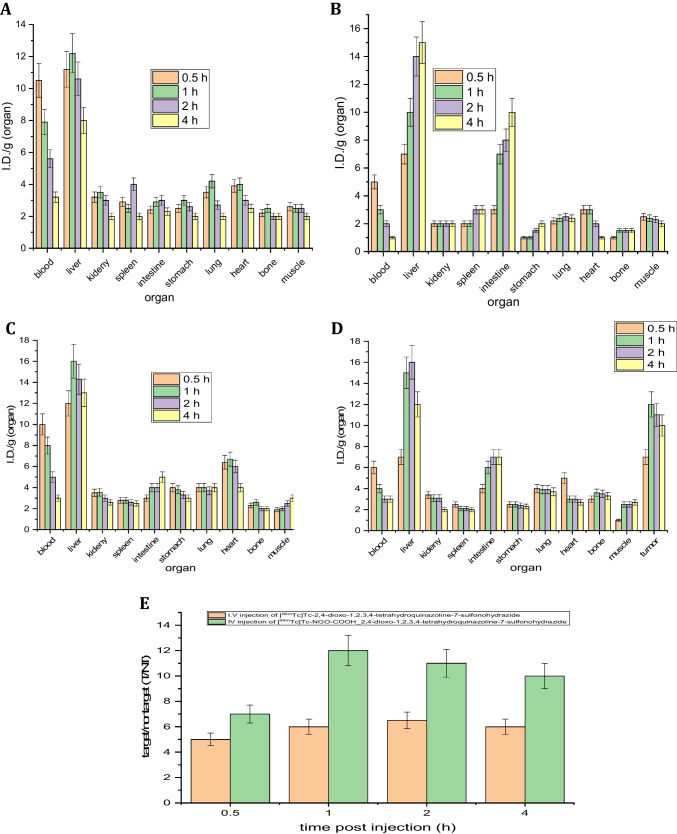
**A** In vivo biodistribution of [^99m^Tc]Tc-2,4-dioxo-1,2,3,4-tetrahydroquinazoline-7-sulfonohydrazide in normal Albino mice at different time intervals post intravenous injection; I.V.(% ID/g). **B** In vivo biodistribution of [^99m^Tc]Tc-NGO-COOH-2,4-dioxo-1,2,3,4-tetrahydroquinazoline-7-sulfonohydrazidein normal Albino mice at different time intervals post intravenous injection; I.V.(% ID/g). **C** In vivo biodistribution of [^99m^Tc]Tc-2,4-dioxo-1,2,3,4-tetrahydroquinazoline-7-sulfonohydrazide in solid tumor-bearing Albino mice at different time intervals post intravenous injection; I.V.(% ID/g). **D** In vivo biodistribution of [^99m^Tc]Tc-NGO-COOH-2,4-dioxo-1,2,3,4-tetrahydroquinazoline-7-sulfonohydrazidenanosheets in solid tumor-bearing Albino mice at different time intervals post intravenous injection; I.V.(% ID/g). **E** T/NT ratio of [^99m^Tc]Tc-2,4-dioxo-1,2,3,4-tetrahydroquinazoline-7-sulfonohydrazide and [^99m^Tc]Tc-NGO-COOH-2,4-dioxo-1,2,3,4-tetrahydroquinazoline-7-sulfonohydrazide at different times post I.V. Injection in solid tumor-tumor bearing Albino mice

The overall approach for studying the biodistribution pattern of the radiolabeld complex is outline in Fig. 13. The biodistribution of ^99m^Tc-3 following administration in normal mice is presented in Fig. 14A. As can be observed, the initial blood uptake of ^99m^Tc -3 was high (10.5 ± 1.4%ID/g) at 0.5 h p.i. The radioactivity was cleared gradually with time from the blood reaching 3.2 ± 0.4%ID/g at 4 h p.i. The observed radioactivity uptake in the liver was relatively high starting with 11 ± 1.7%ID/g (0.5 h p.i) and reached 12 ± 1.2%ID/g (1 h p.i). The liver uptake started declining after 1 h and reached its lowest measured value at 4 h p.i (8 ± 1.3%ID/g). On the contrary, the kidney showed moderate uptake of radioactivity when compared to the liver which indicated that the metabolism and excretion of ^99m^Tc -3 is mainly through hepatobiliary pathway. The accumulation of radioactivity at the stomach was not high which indicated that the complex is stable in vivo and no radiolysis occurred. localization of ^99m^Tc-3 at tumor site was High and rapid (0.5 p.i) with uptake of 5.0 ± 0.40%ID/g and the uptake continued increasing to reach 6.5 ± 0.3%ID/g after 2 h then 6.0 ± 0.4%ID/g at 4 h p.i (Fig. 14C). The fast uptake and prolonged retention of ^99m^Tc -3 at tumor site indicated that compound **3** has the potential to be a targeted therapeutic agent.

#### Biodistribution of [^99m^Tc] Tc-NGO-COOH-3

The observed radioactivity in the blood was much lower than that in case of ^99m^Tc -3 with uptake of 5.0 ± 0.5%ID/g at 30 min. after injection and reaching 1 ± 0.1%ID/g at 4 h after injection (Fig. 14B) which indicated fast distribution of the nano-system throughout the body. Also, the initial liver uptake at 30 min. after injection. (7.0 ± 0.7%ID/g) was lower than that of ^99m^Tc -3 at the same time point (11.0 ± 1.7%ID/g). However, after 4 h p.i the uptake reached 15.0 ± 1.5%ID/g 4 h. This high uptake can be explained by the fact that; NGO-COOH conjugates hydrodynamic diameter studied in this analysis is considerably larger than the cutoff for renal filtration (5 nm), thus, hepatobiliary pathway is expected to be the major route for clearance (Fig. 14B) [67]. For ^99m^Tc-NGO-COOH-3, the tumor uptake was 7.0 ± 0.70%ID/g at 0.5 h which was nearly duplicated after 1 h p.i. to reach 12.0 ± 1.2%ID/g. The uptake remained steady for 4 h (Fig. 14D). The pattern of tumor uptake of ^99m^Tc-NGO-COOH-3 showed high uptake, excellent accumulation and retention especially when compared with ^99m^Tc-olaparib (3.2 ± 0.36%ID/g, 2 h p.i). Comparing the tumor uptake of ^99m^Tc -3 and ^99m^Tc-NGO-COOH-3, the later complex clearly showed higher uptake of radioactivity at all time points. High uptake with long retention and two folds increased uptake of the radioactivity in short time assured that; the designed nanosheets played a major role in selective accumulation of the radioactivity at tumor site. Also, the tumor uptake was further augmented by EPR phenomena [9, 68–71]. In addition, tumor tissues lose a disposal lymphatic system, which enhanced the retention of ^99m^Tc-NGO-COOH-3. Finally, nanosheets being coated with hydrophilic group like carboxylic group help to prevent the ReticuloEndothelial System (RES) mechanism by reducing in vivo adsorption [16, 72, 73]. The RES is essentially responsible for clearance of Nanoparticles from the biological system.

#### Selective localization and targeting of tumor tissue

Target/ non-target ratio is often utilized to express selective localization degree of radiopharmaceuticals. In this study, the target (tumor muscle) uptake to non-target uptake (contralateral normal muscle) was evaluated for the tumor uptake of both ^99m^Tc-3 and ^99m^Tc-NGO-COOH-3. The most effective techniques for reducing RES absorption involve stabilizing nanosheets with hydrophilic groups [33, 73–76]. Phagocytes are unable to infiltrate because a hydrated water barrier provides considerable steric hindrance, which remained consistent during its in vivo tests. The high target/nontarget ratio, which was 9 after 2 h post-injection, is a benefit of the suggested radiolabeled nano-system (^99m^Tc- NGO-COOH). The greatest T/NT ratio for previously published radiolabeled nano-systems was 3.7 ± 0.45–7 ± 0.5 [15, 74, 75]. While NGO T/NT ratio was 2 ± 0.5 as reported [76].

## Conclusion

Nanographene oxide modified derivative (NGO) NGO-COOH nanosheets have overexpressed surface area and capacity to carry a huge payload so it is a novel nano-sheet material that have potential pathways to tumors in vivo. NGO-COOH holds promise as a various scaffold material for the development of molecular imaging probes while 2,4-dioxo-1,2,3,4-tetrahydroquinazoline-7-sulfonohydrazide (3) is promising inhibitors for PARP-1 enzyme. So, combination of both nanomaterial and the PRAP-1 inhibitor and labeling them by ^99m^Tc and also the exploitation of the advantage of the active targeting of the nano-system to tumor cells give a high potential opportunity to have a novel potent drug that may start a new era in tumor theragnosis.

### Supplementary Information

Supplementary Figure 1 Low resolution image (PNG 518 kb)

High resolution image (TIF 93.3 kb)

Supplementary Figure 2 Low resolution image (PNG 94.9 kb)

High resolution image (TIF 18.1 kb)

Supplementary Figure 3 Low resolution image (PNG 5.44 kb)

High resolution image (TIF 4.93 kb)

Supplementary Figure 4 Low resolution image (PNG 80.4 kb)

High resolution image (TIF 17.1 kb)

Supplementary Figure 5 Low resolution image (PNG 86.8 kb)

High resolution image (TIF 17.6 kb)

Supplementary Figure 6 Low resolution image (PNG 86.5 kb)

High resolution image (TIF 17.6 kb)

Supplementary Figure 7 Low resolution image (PNG 94.6 kb)

High resolution image (TIF 18.1 kb)

Supplementary Figure 8 Low resolution image (PNG 94.7 kb)

High resolution image (TIF 18.1 kb)

## Data Availability

Data will be available.
