# Supplementary figures and images for: Multi-functionalization of reduced graphene oxide nanosheets for tumor theragnosis: Synthesis, characterization, enzyme assay, in-silico study, radiolabeling and in vivo targeting evaluation
Source: Daru. 2023 Dec 11;32(1):77–95. doi: 10.1007/s40199-023-00487-7 (PMC11087444; doi:10.1007/s40199-023-00487-7)

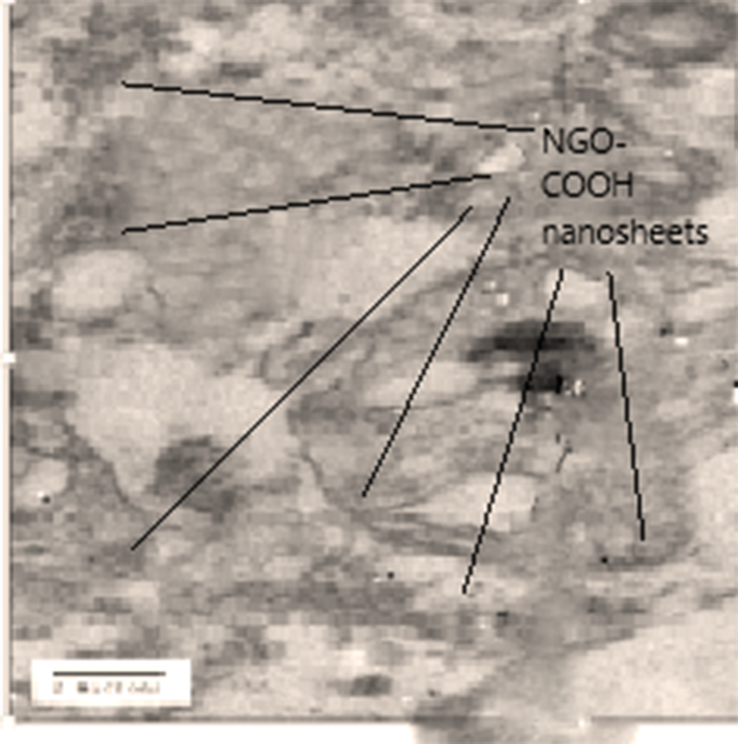

Supplement: Supplementary file 1 — Low resolution image (PNG 518 kb) [file 40199_2023_487_Fig15_ESM.png]

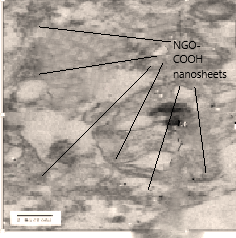

Supplement: Supplementary file 2 — High resolution image (TIF 93.3 kb) [file 40199_2023_487_MOESM1_ESM.tif]

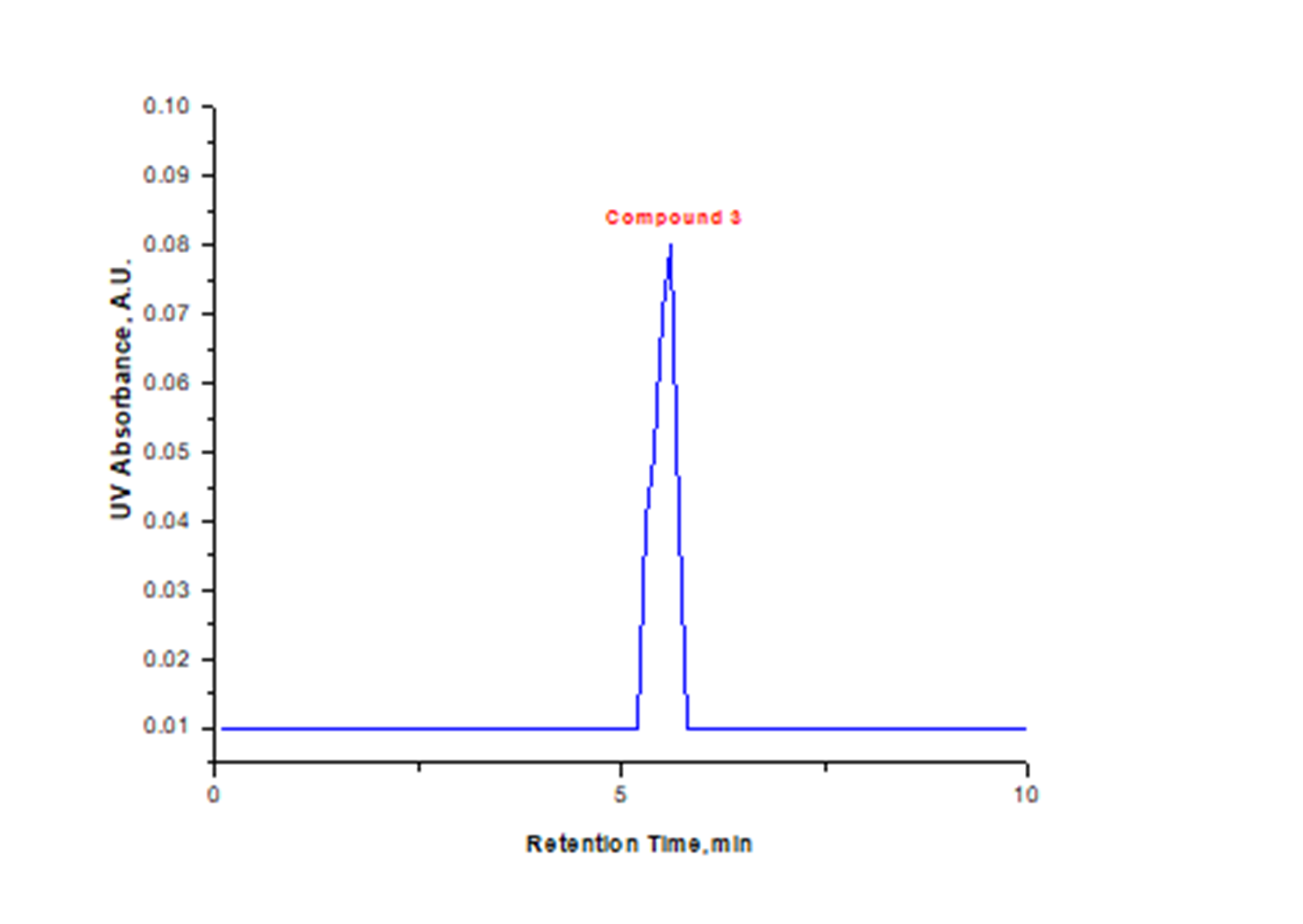

Supplement: Supplementary file 3 — Low resolution image (PNG 94.9 kb) [file 40199_2023_487_Fig16_ESM.png]

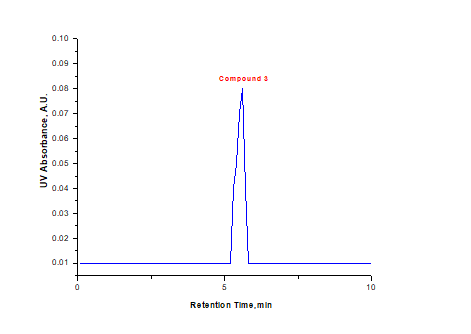

Supplement: Supplementary file 4 — High resolution image (TIF 18.1 kb) [file 40199_2023_487_MOESM2_ESM.tif]

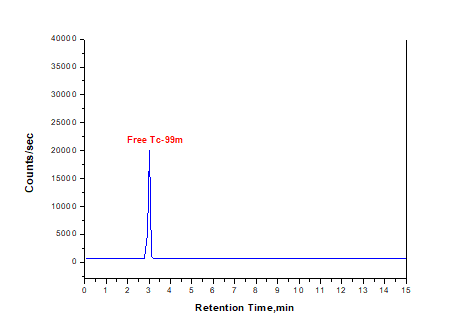

Supplement: Supplementary file 5 — Low resolution image (PNG 5.44 kb) [file 40199_2023_487_Fig17_ESM.png]

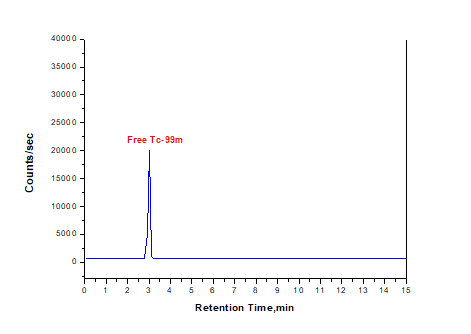

Supplement: Supplementary file 6 — High resolution image (TIF 4.93 kb) [file 40199_2023_487_MOESM3_ESM.tif]

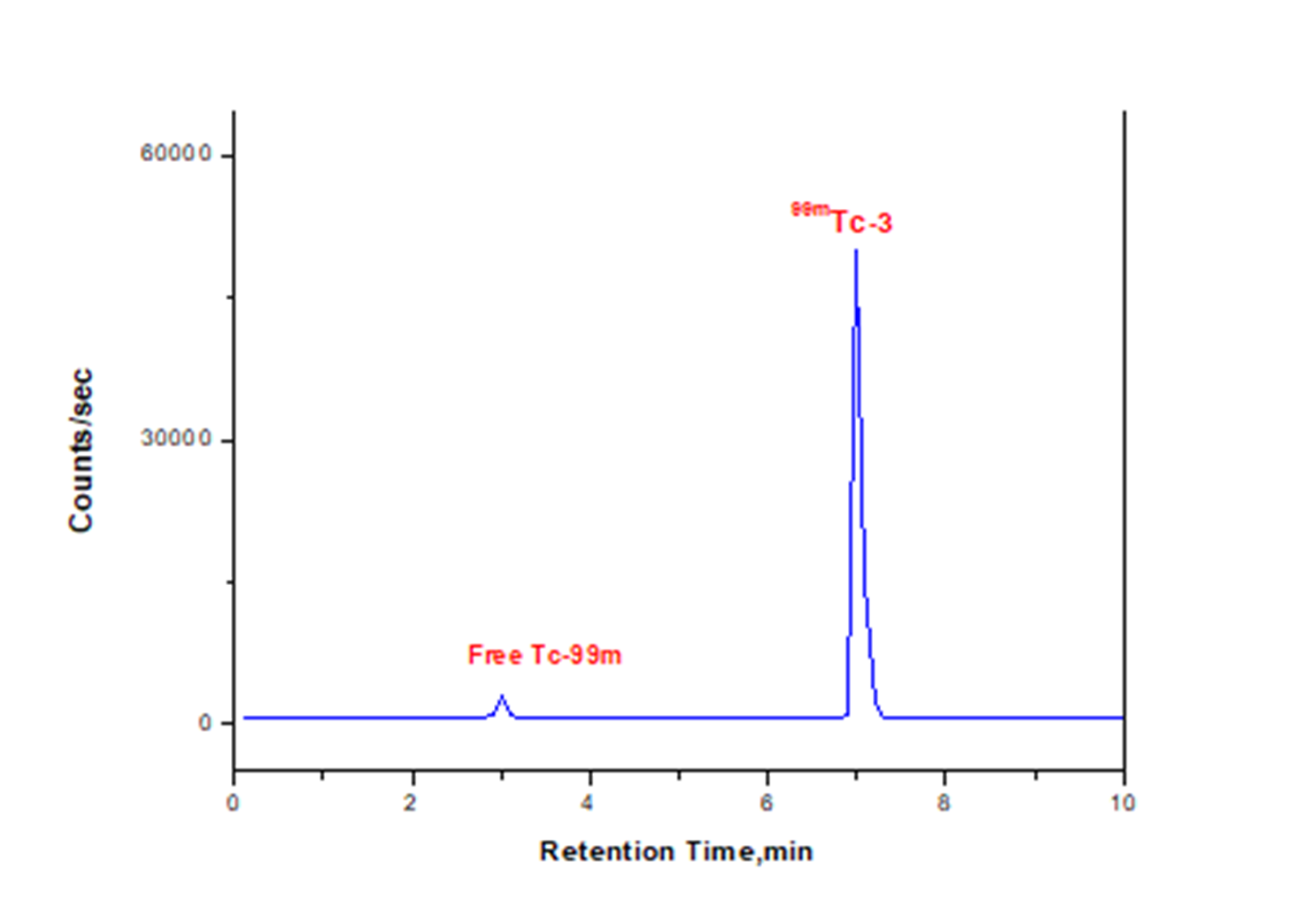

Supplement: Supplementary file 7 — Low resolution image (PNG 80.4 kb) [file 40199_2023_487_Fig18_ESM.png]

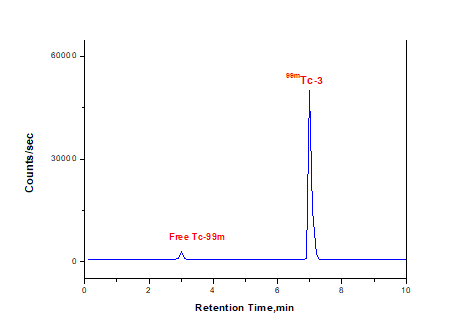

Supplement: Supplementary file 8 — High resolution image (TIF 17.1 kb) [file 40199_2023_487_MOESM4_ESM.tif]

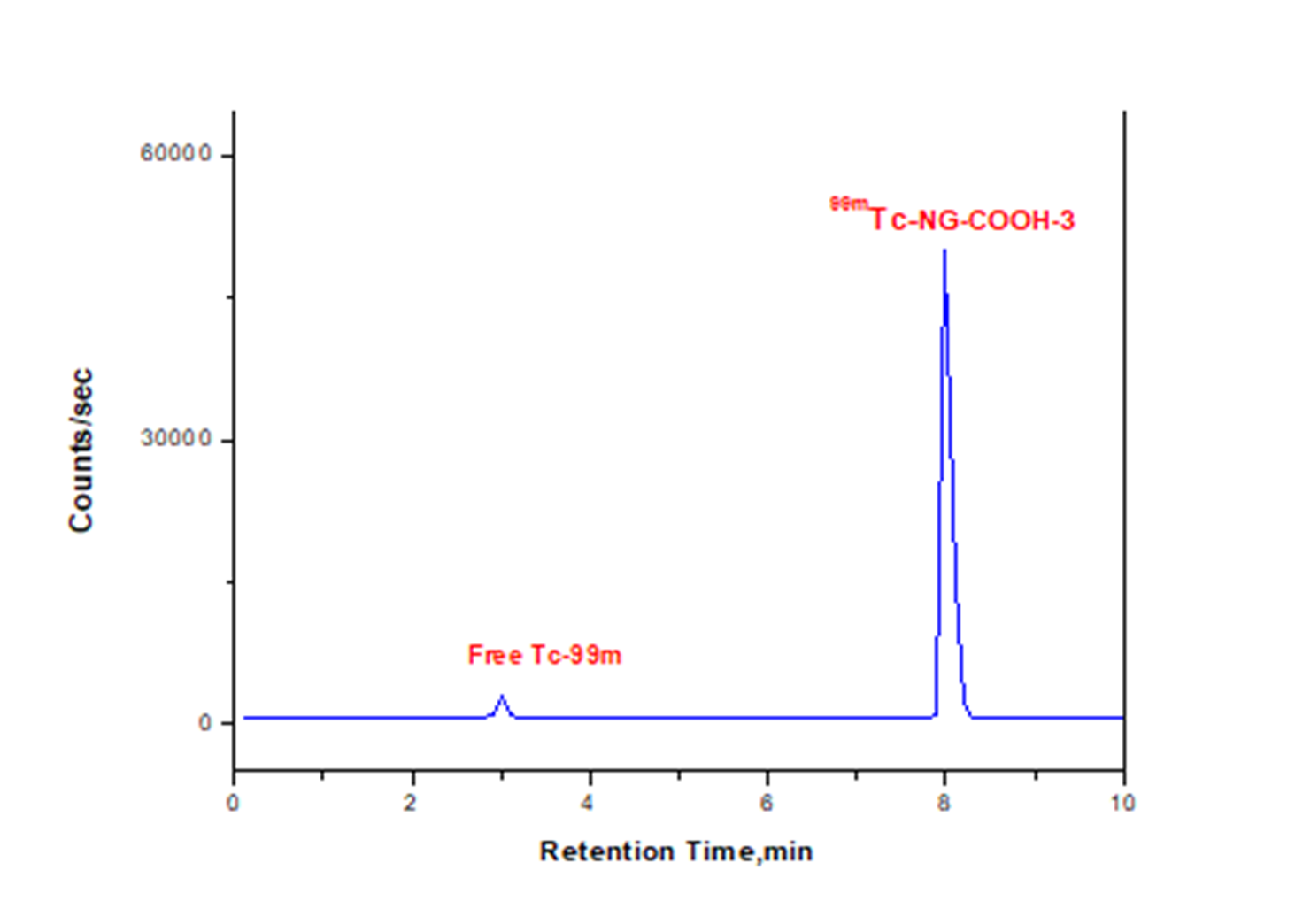

Supplement: Supplementary file 9 — Low resolution image (PNG 86.8 kb) [file 40199_2023_487_Fig19_ESM.png]

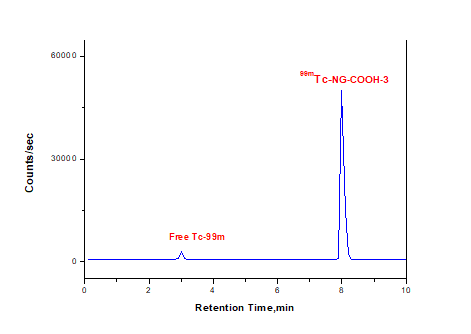

Supplement: Supplementary file 10 — High resolution image (TIF 17.6 kb) [file 40199_2023_487_MOESM5_ESM.tif]

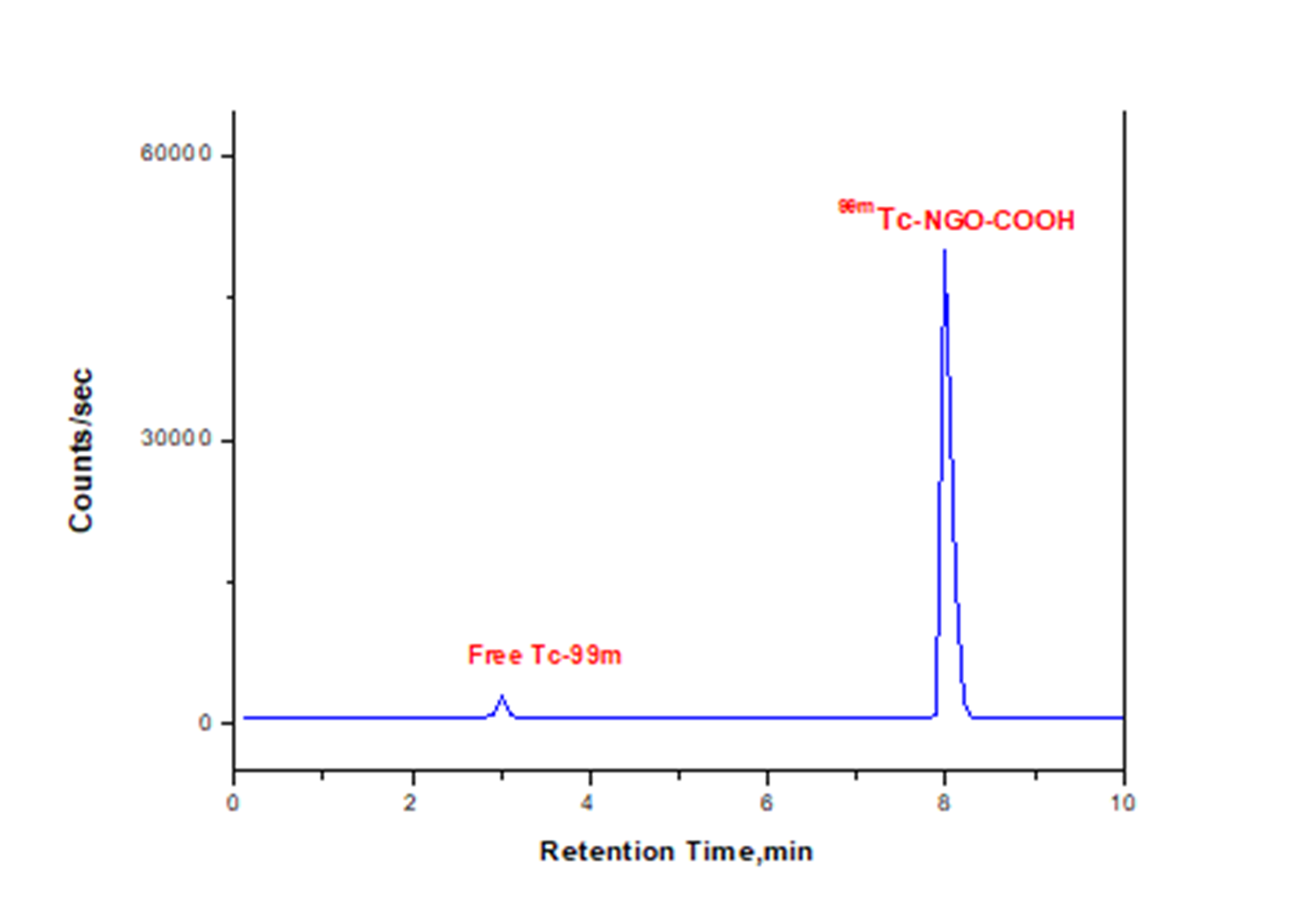

Supplement: Supplementary file 11 — Low resolution image (PNG 86.5 kb) [file 40199_2023_487_Fig20_ESM.png]

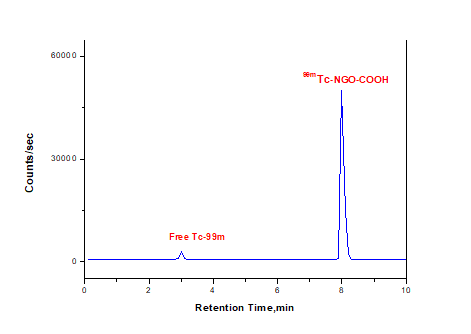

Supplement: Supplementary file 12 — High resolution image (TIF 17.6 kb) [file 40199_2023_487_MOESM6_ESM.tif]

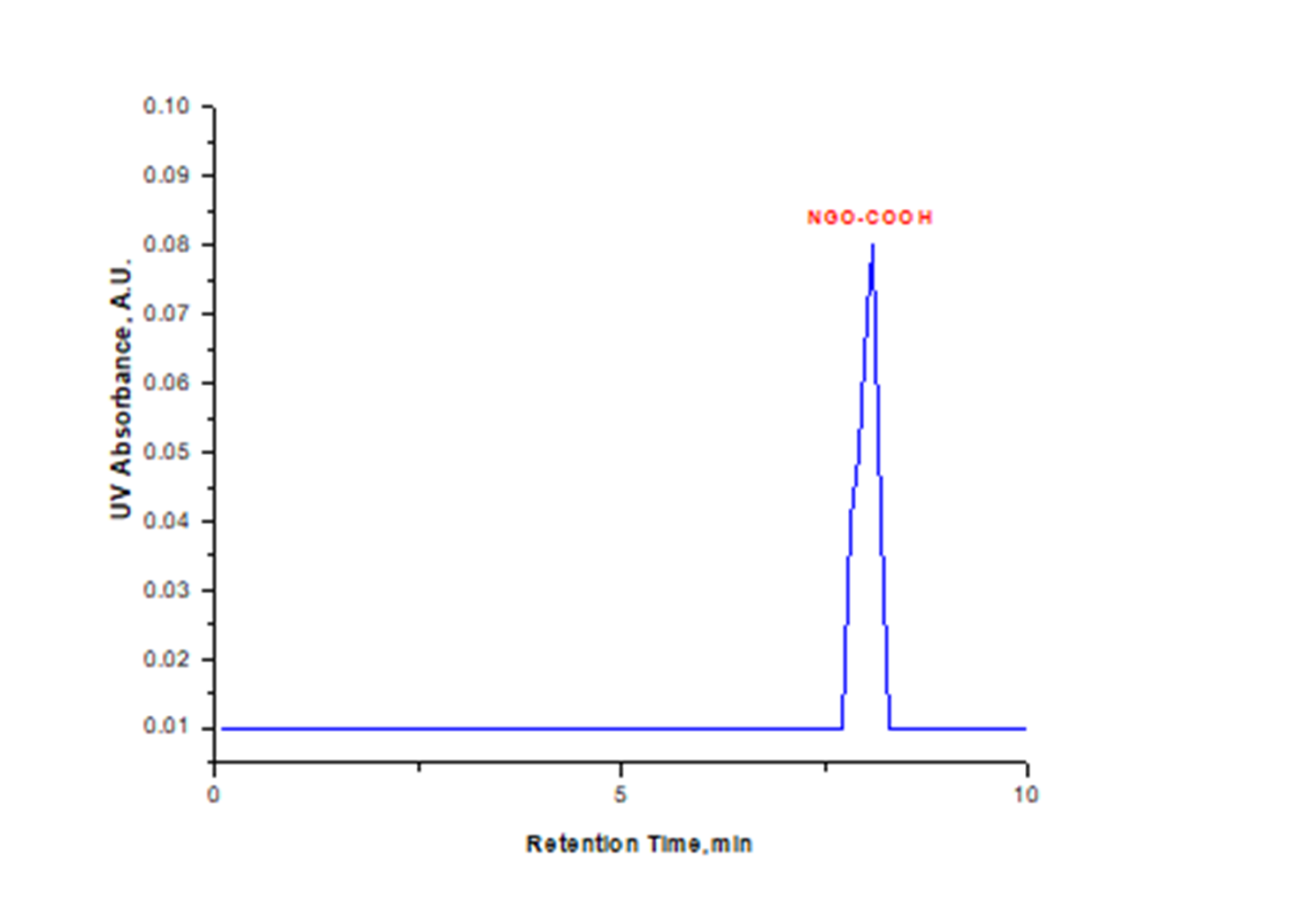

Supplement: Supplementary file 13 — Low resolution image (PNG 94.6 kb) [file 40199_2023_487_Fig21_ESM.png]

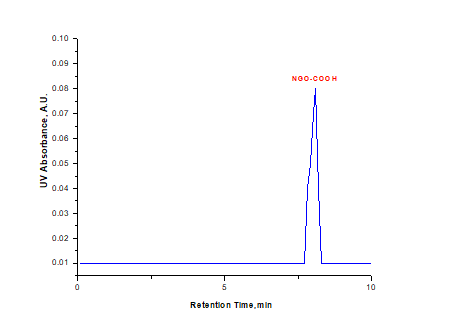

Supplement: Supplementary file 14 — High resolution image (TIF 18.1 kb) [file 40199_2023_487_MOESM7_ESM.tif]

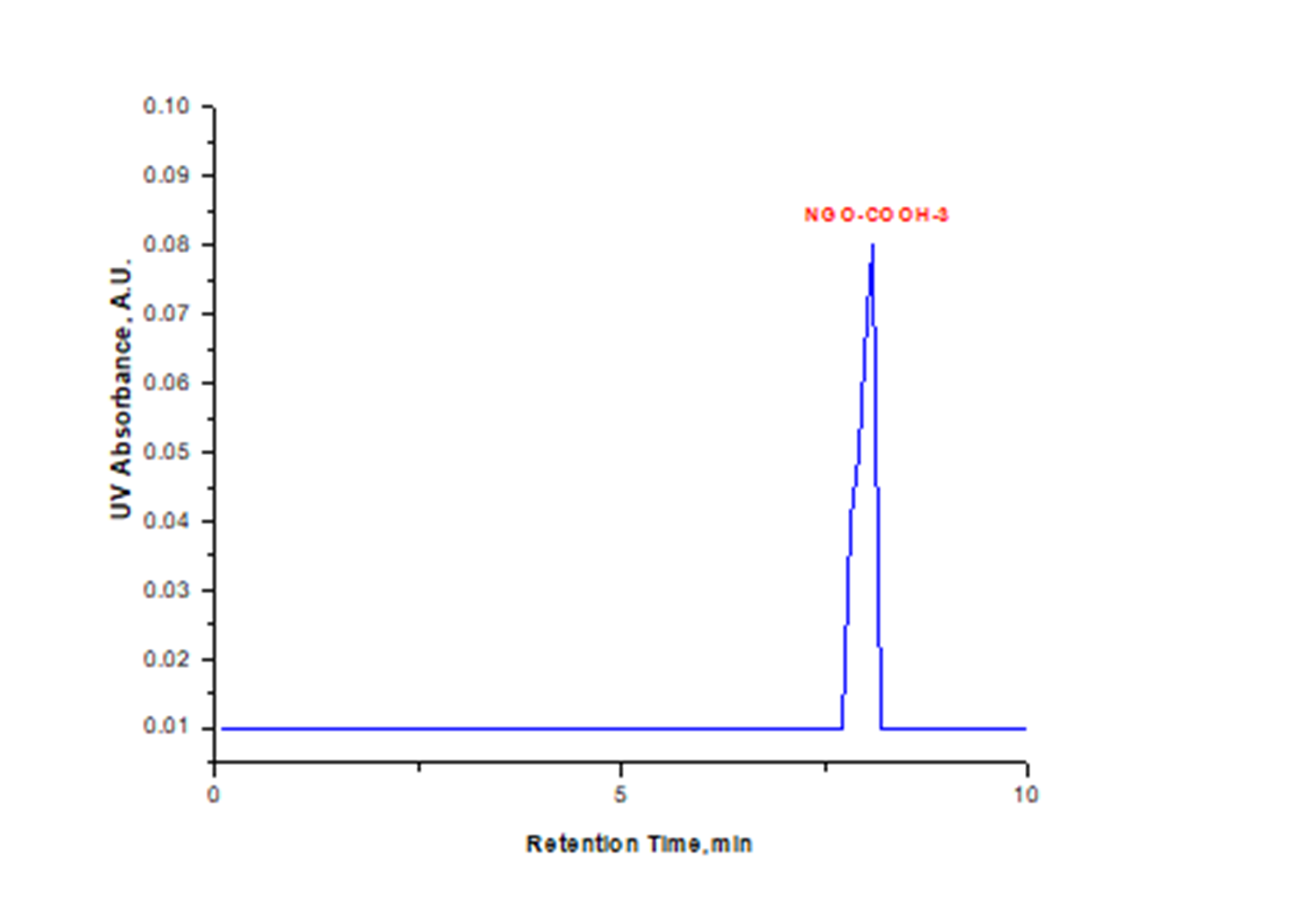

Supplement: Supplementary file 15 — Low resolution image (PNG 94.7 kb) [file 40199_2023_487_Fig22_ESM.png]

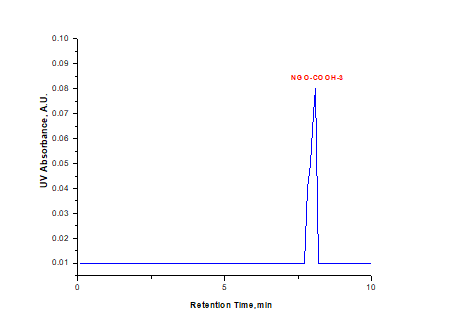

Supplement: Supplementary file 16 — High resolution image (TIF 18.1 kb) [file 40199_2023_487_MOESM8_ESM.tif]
